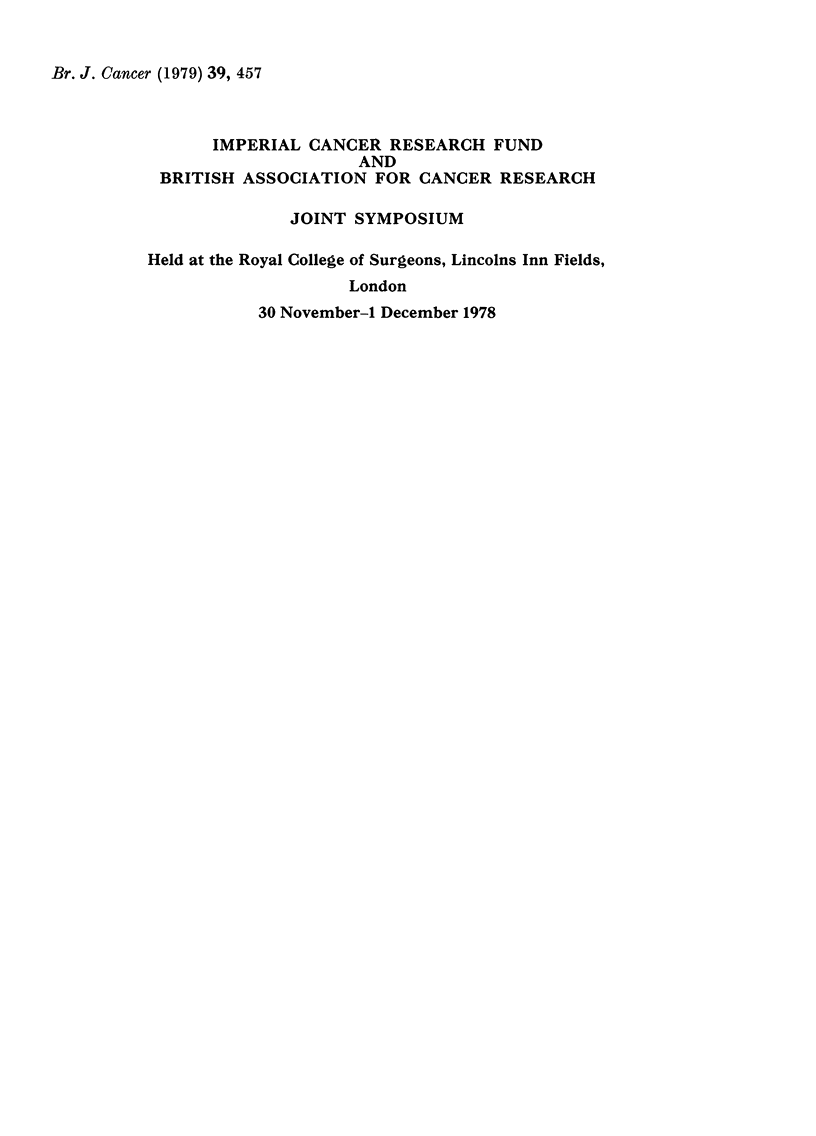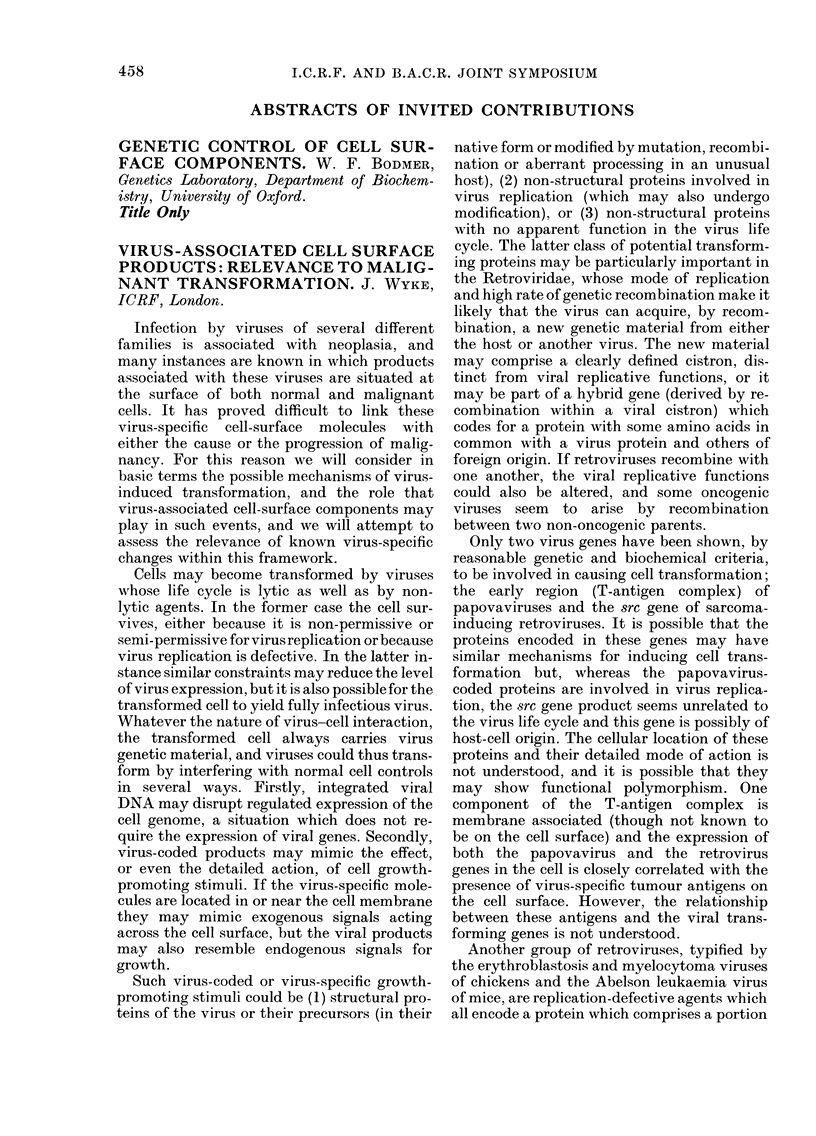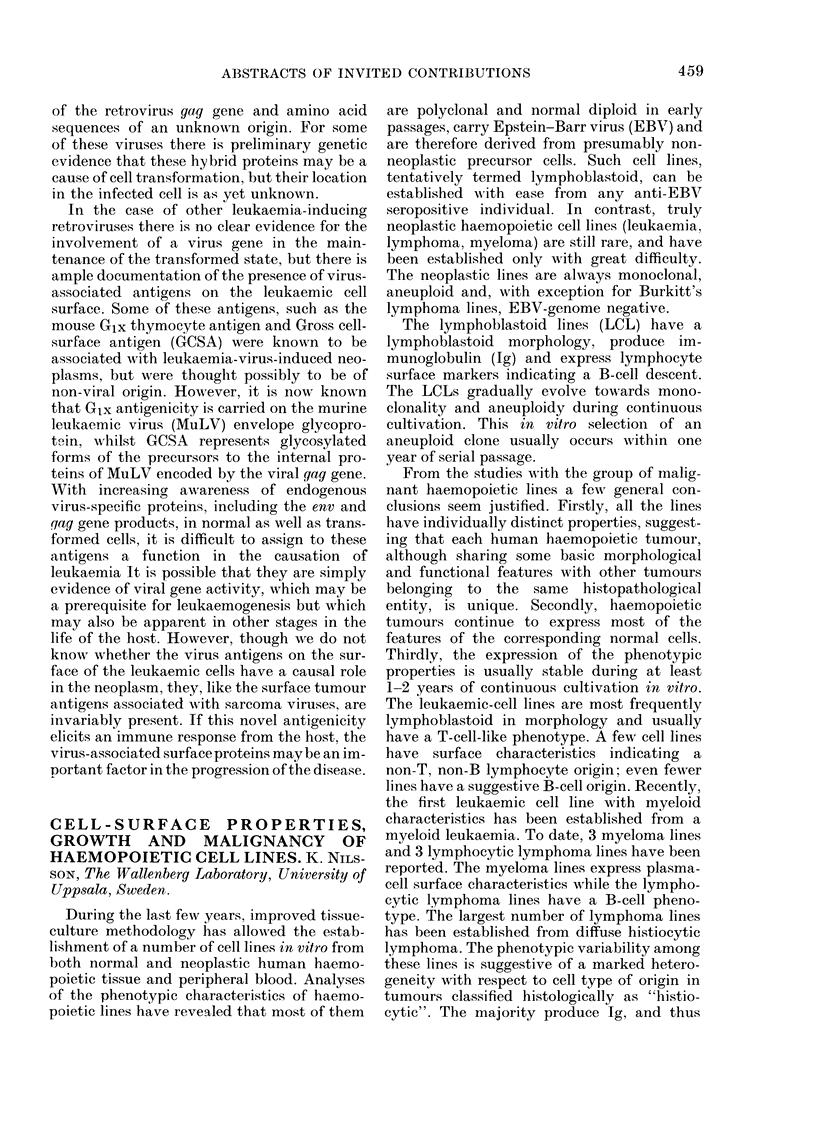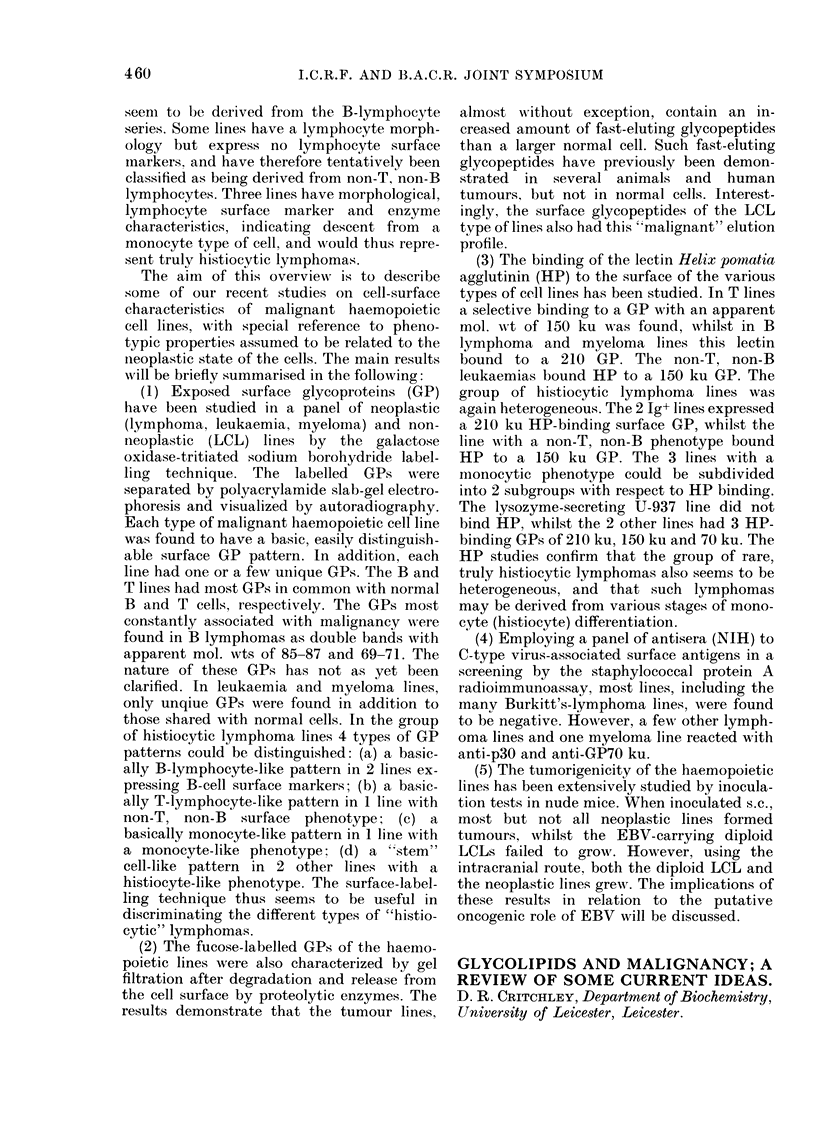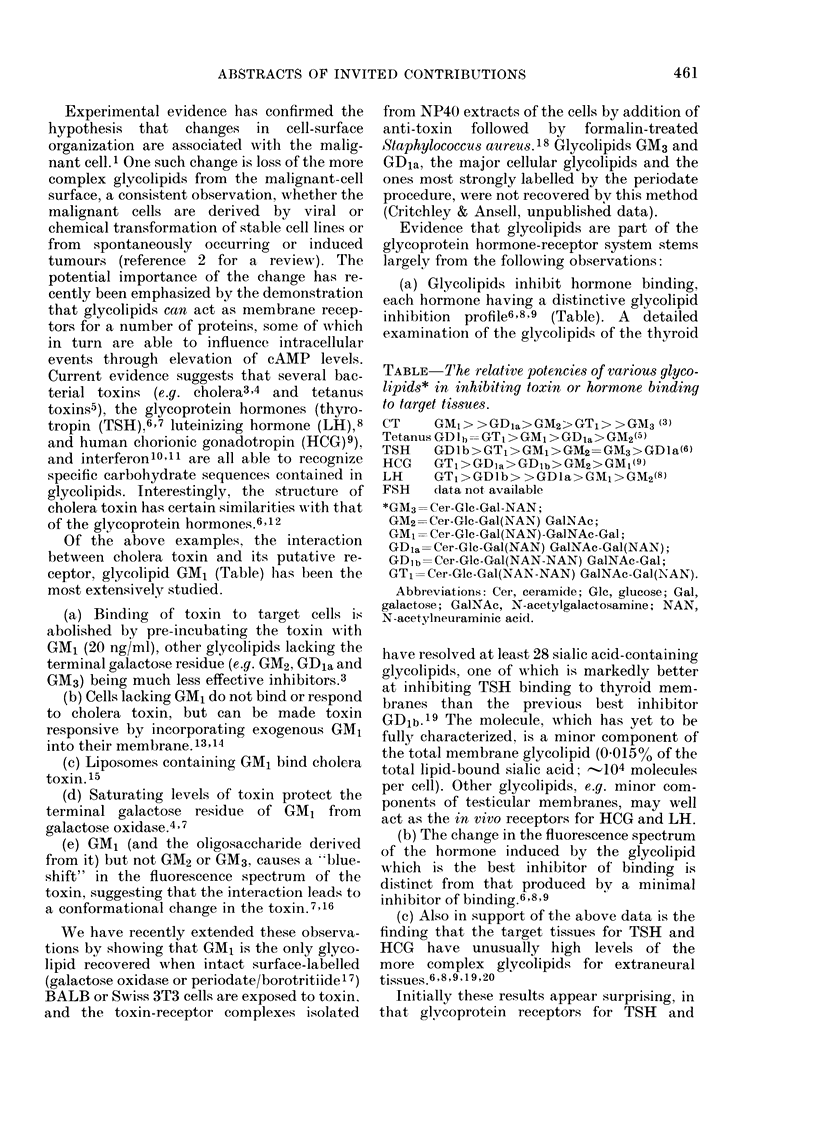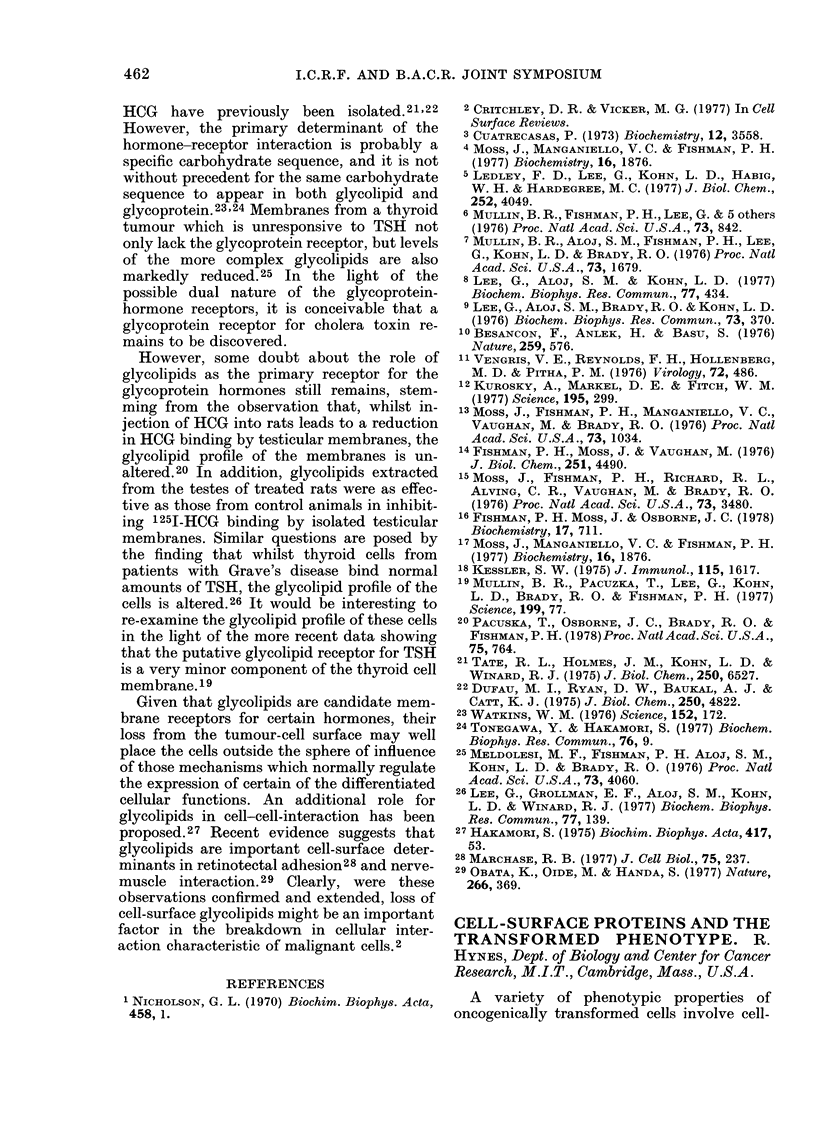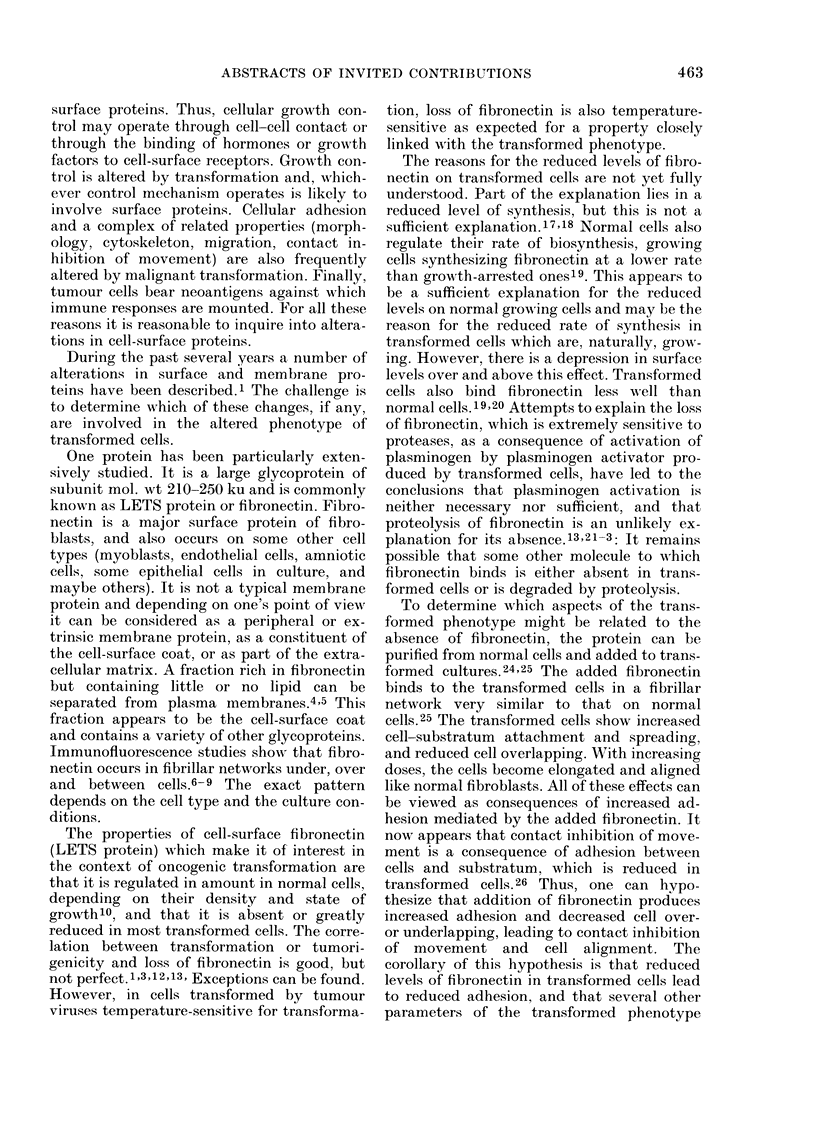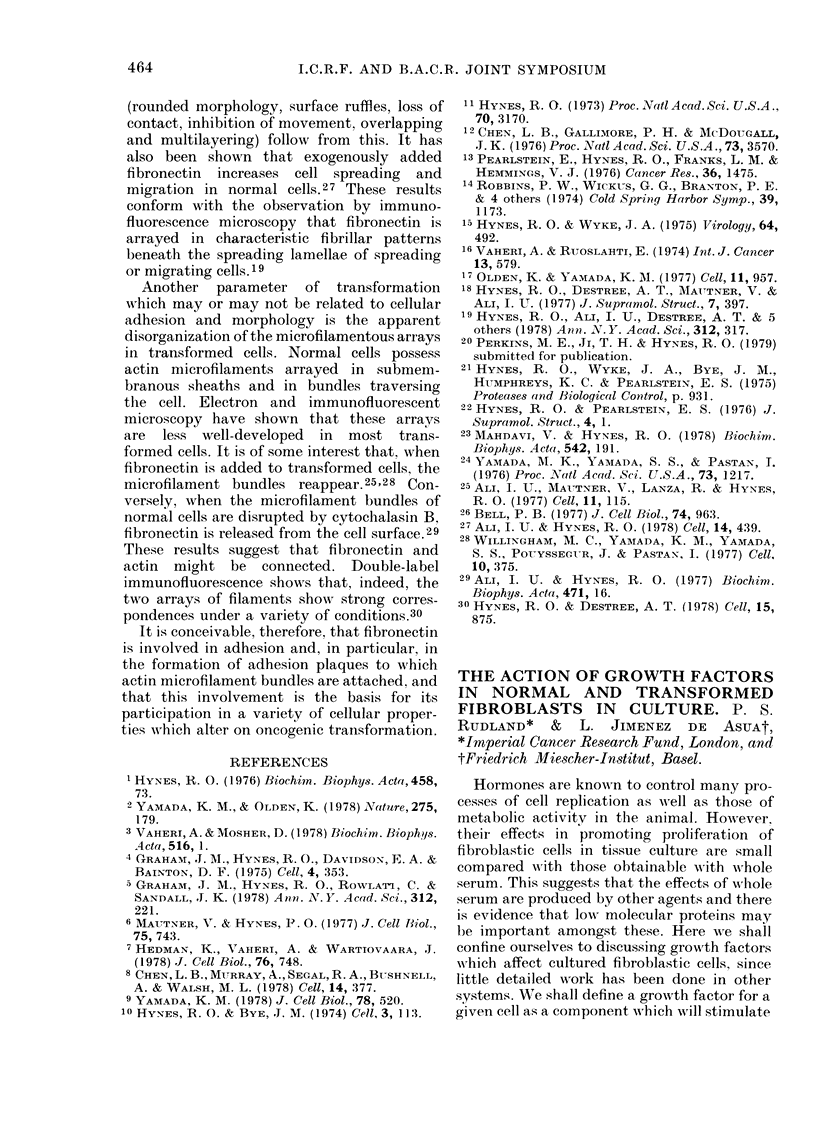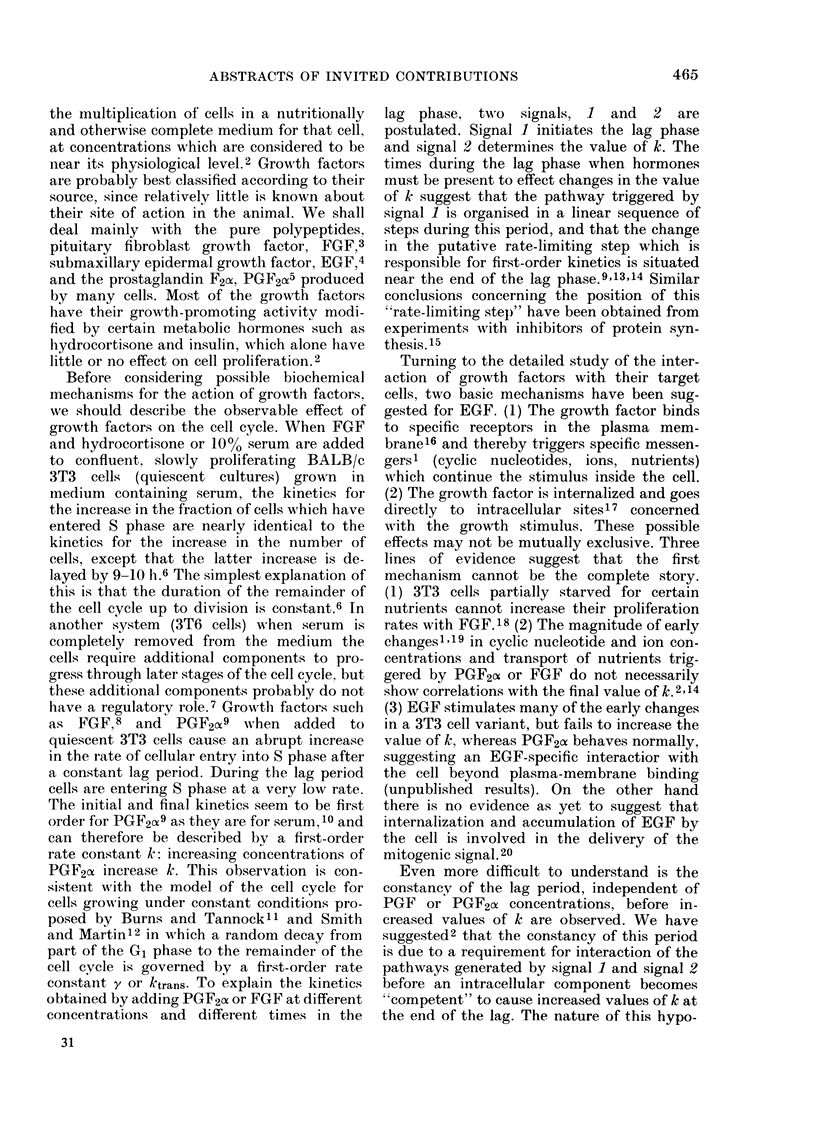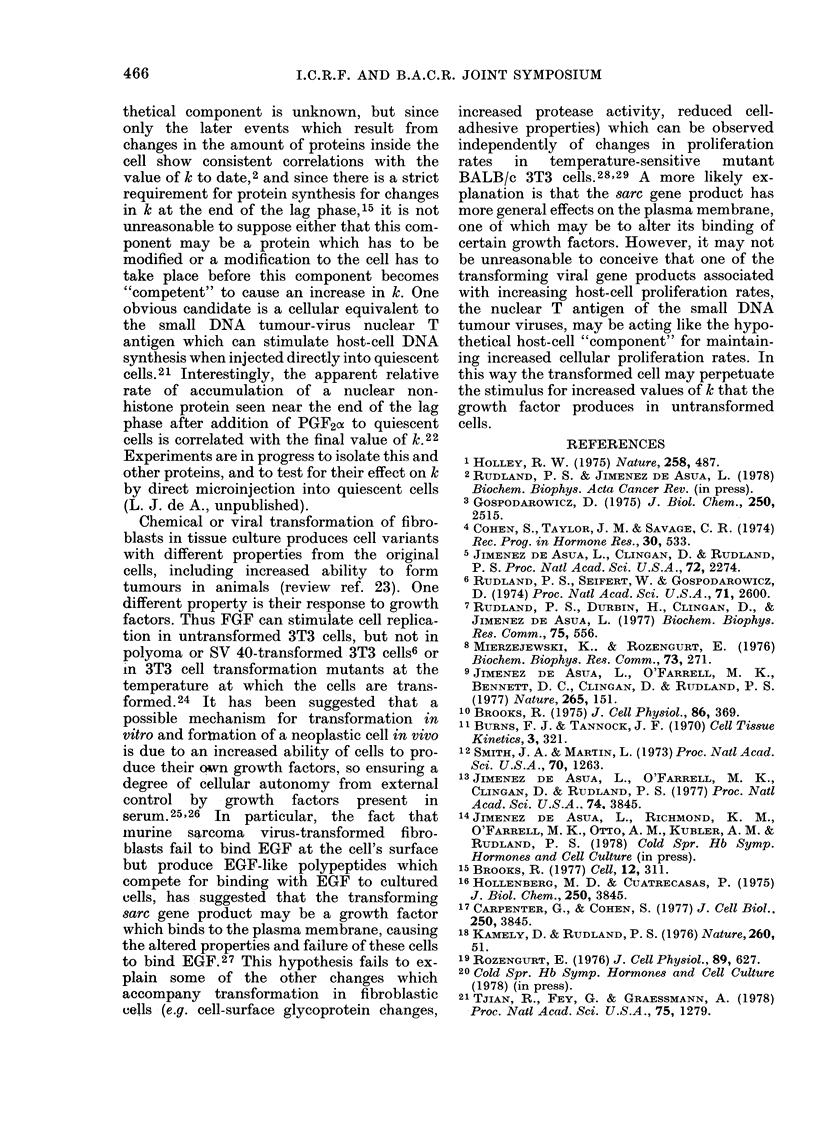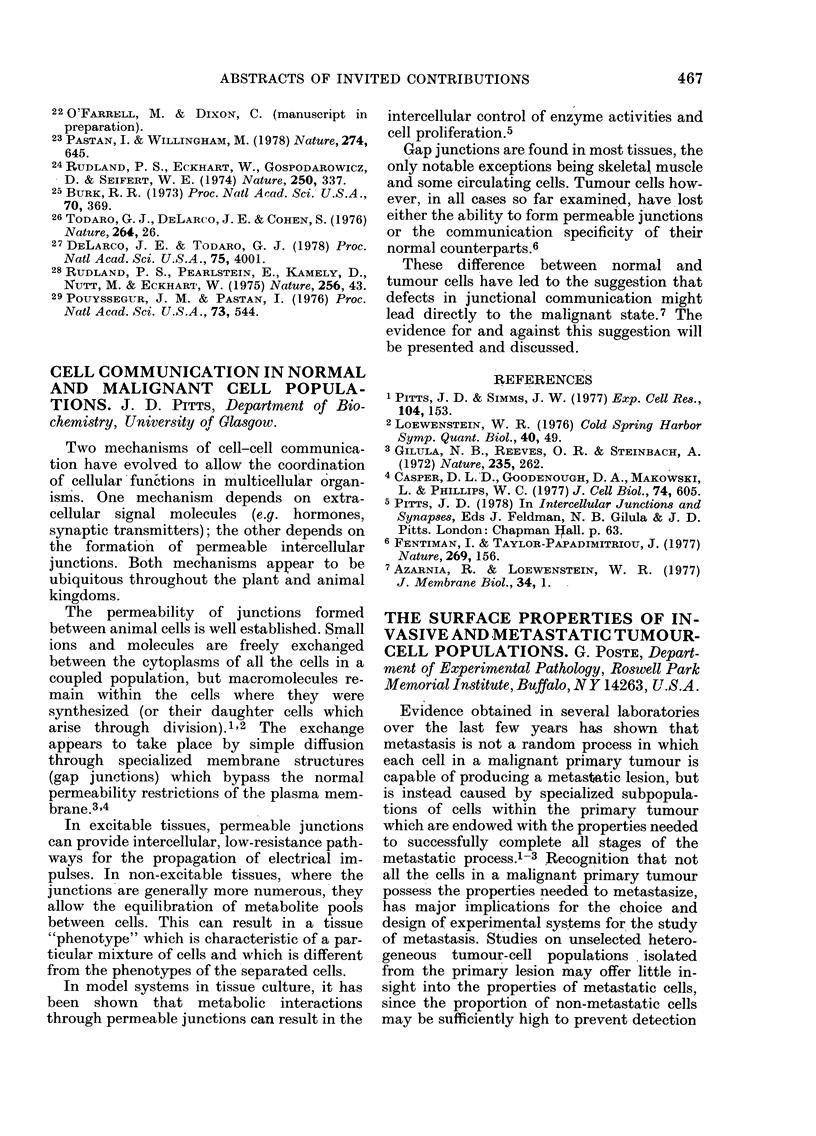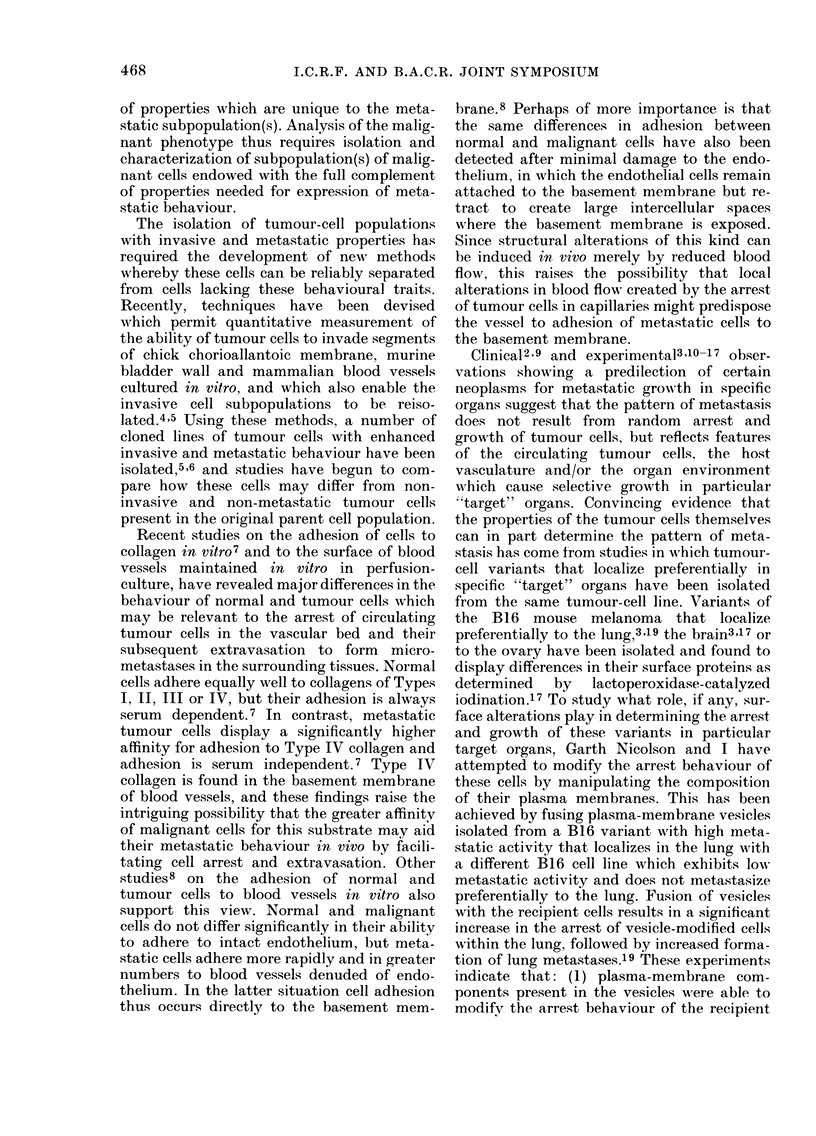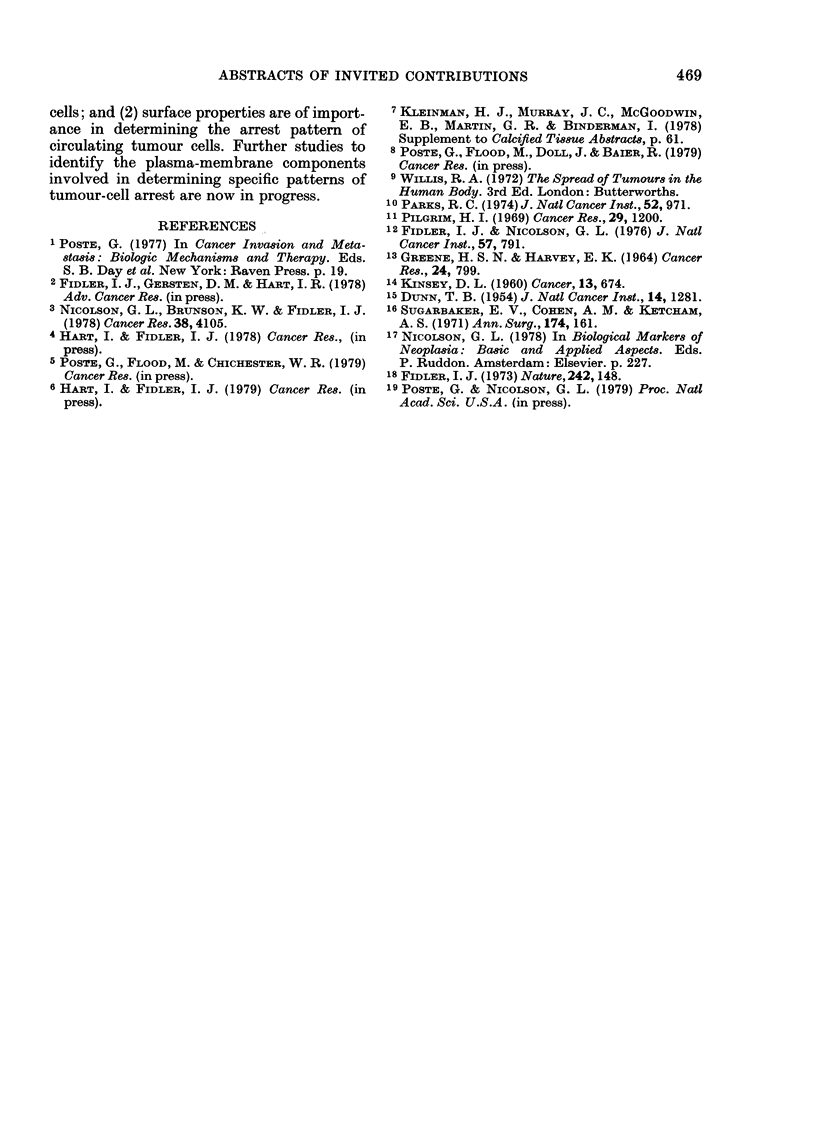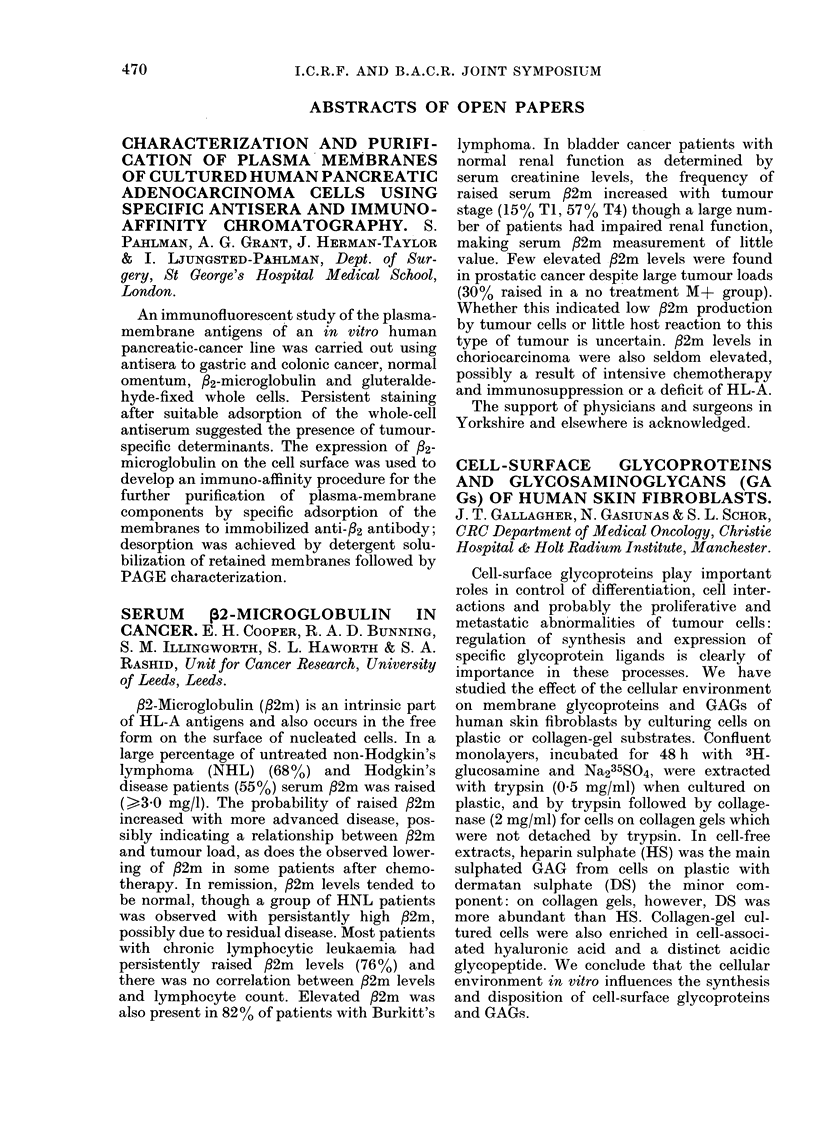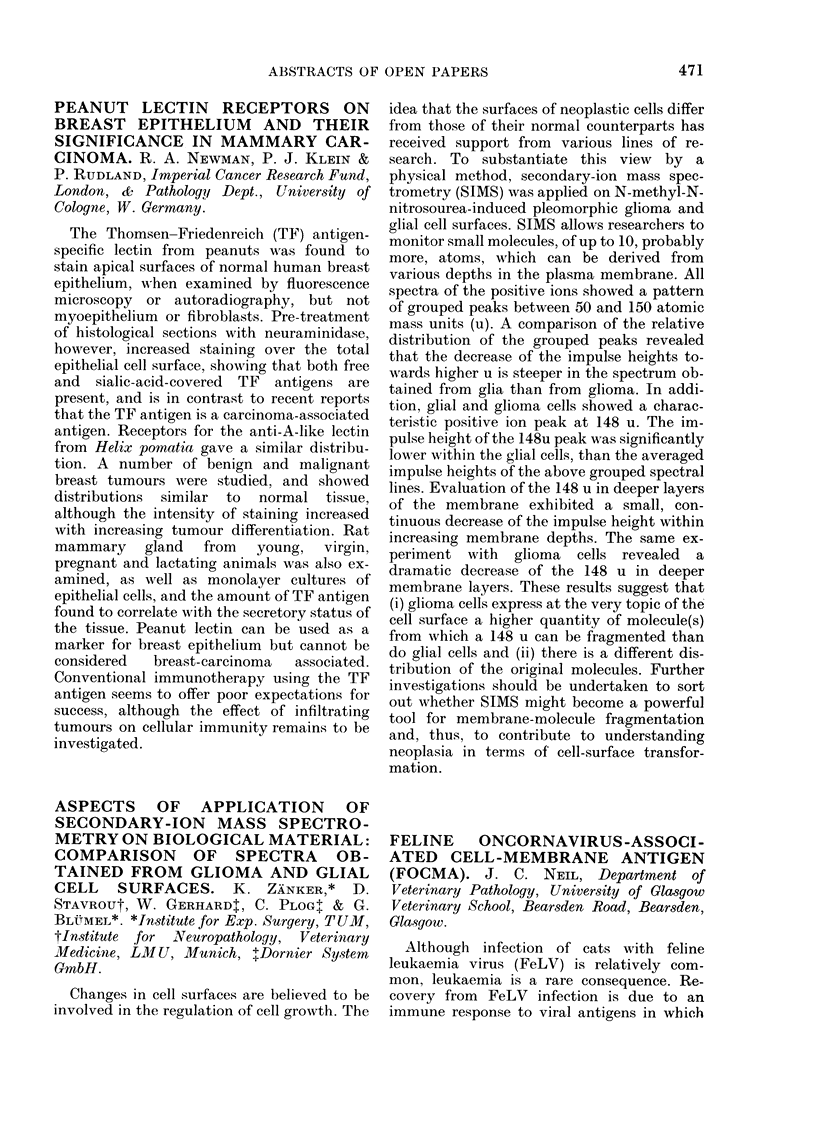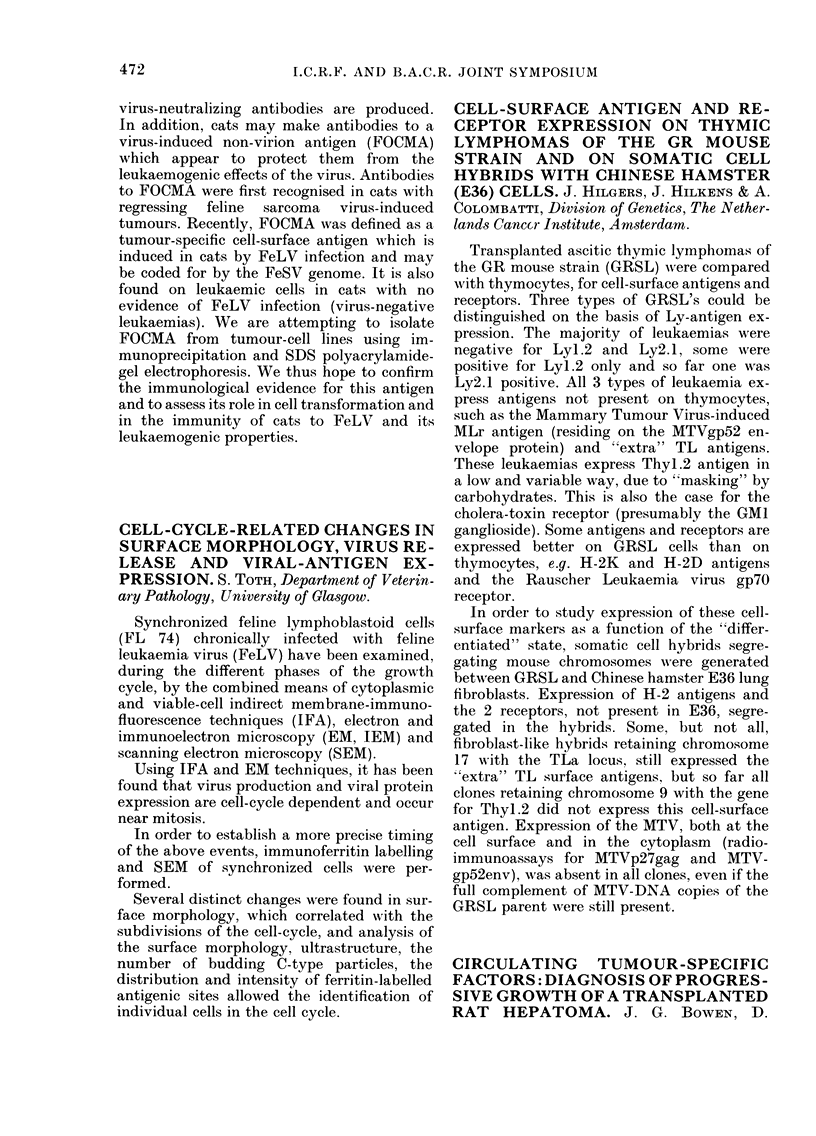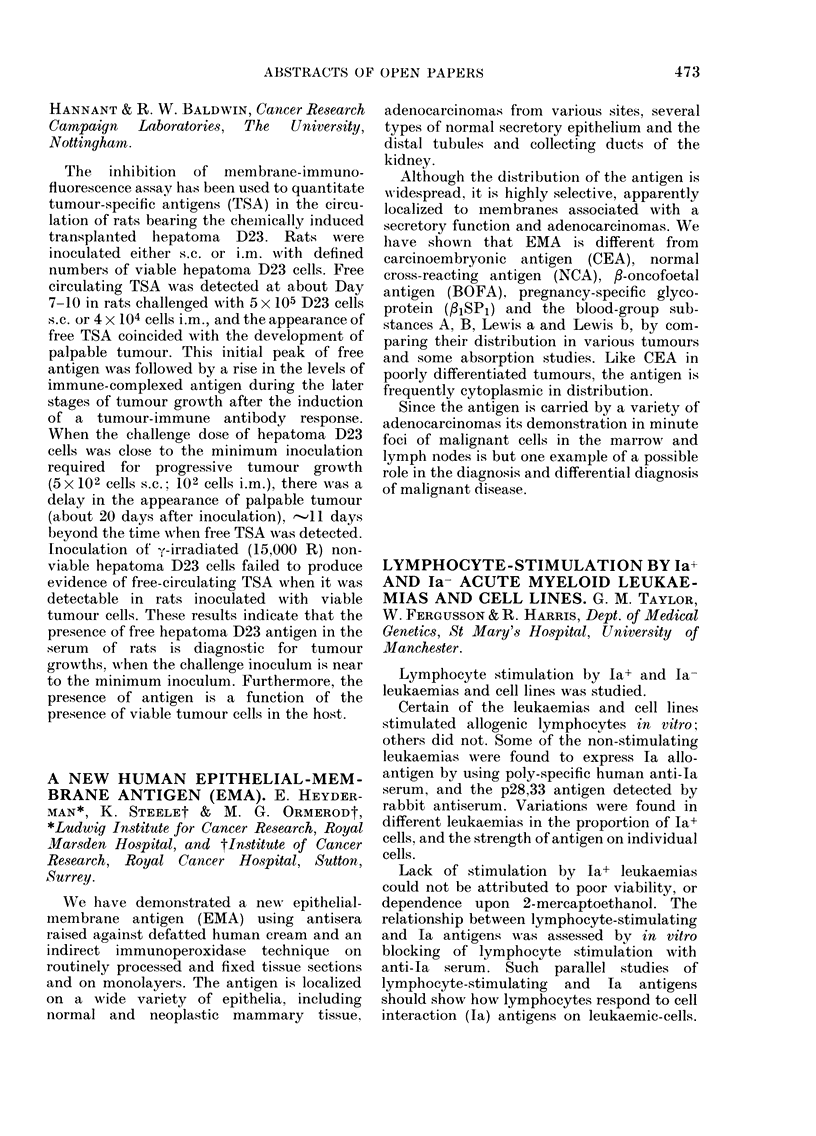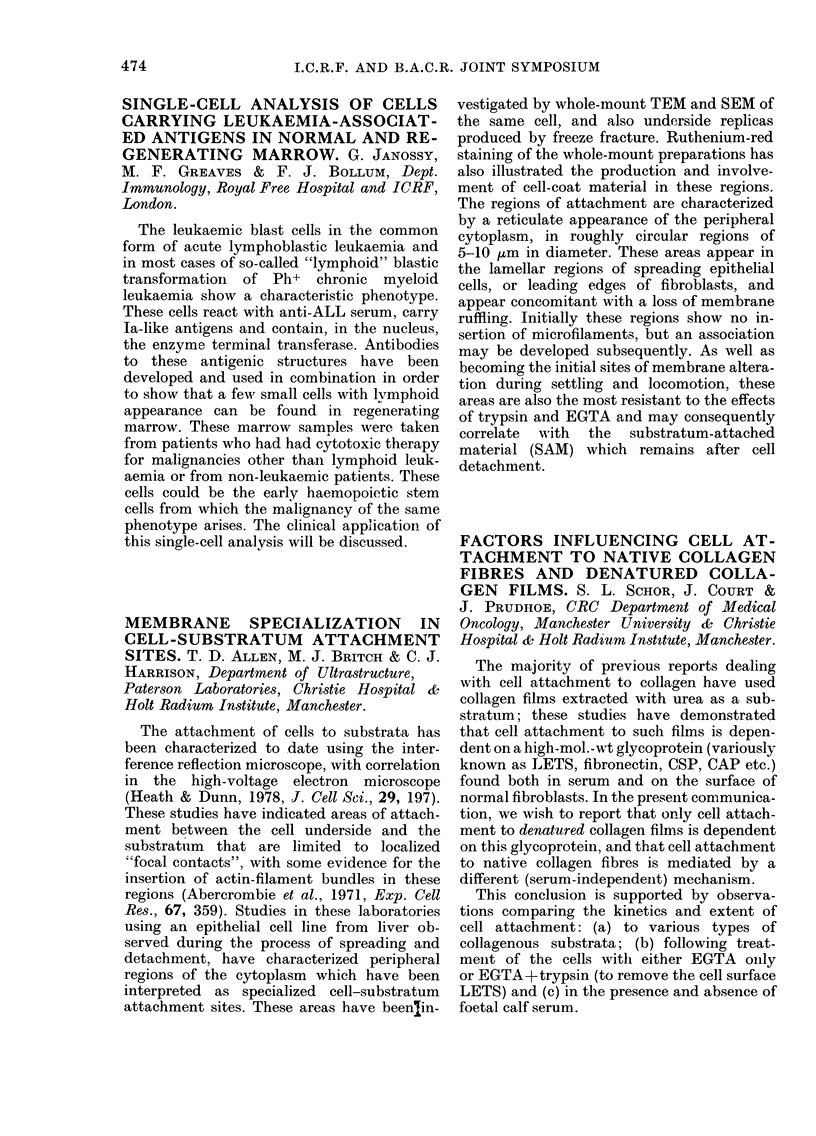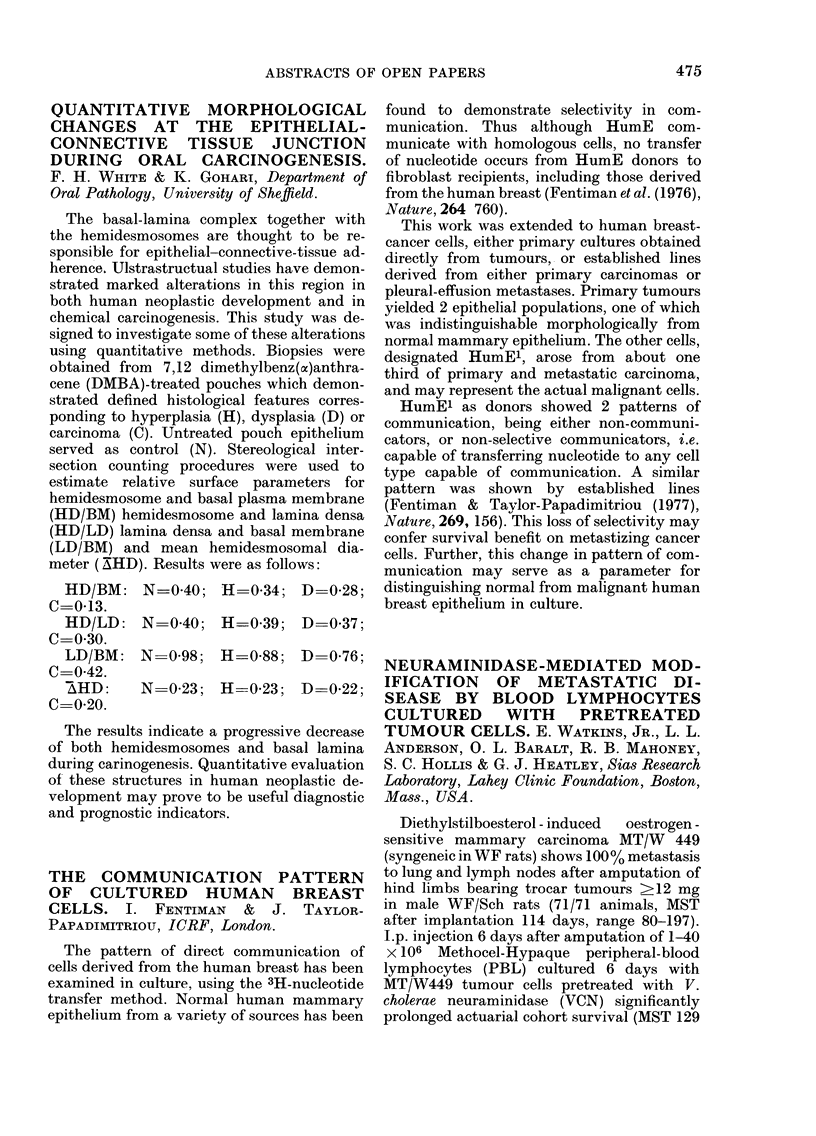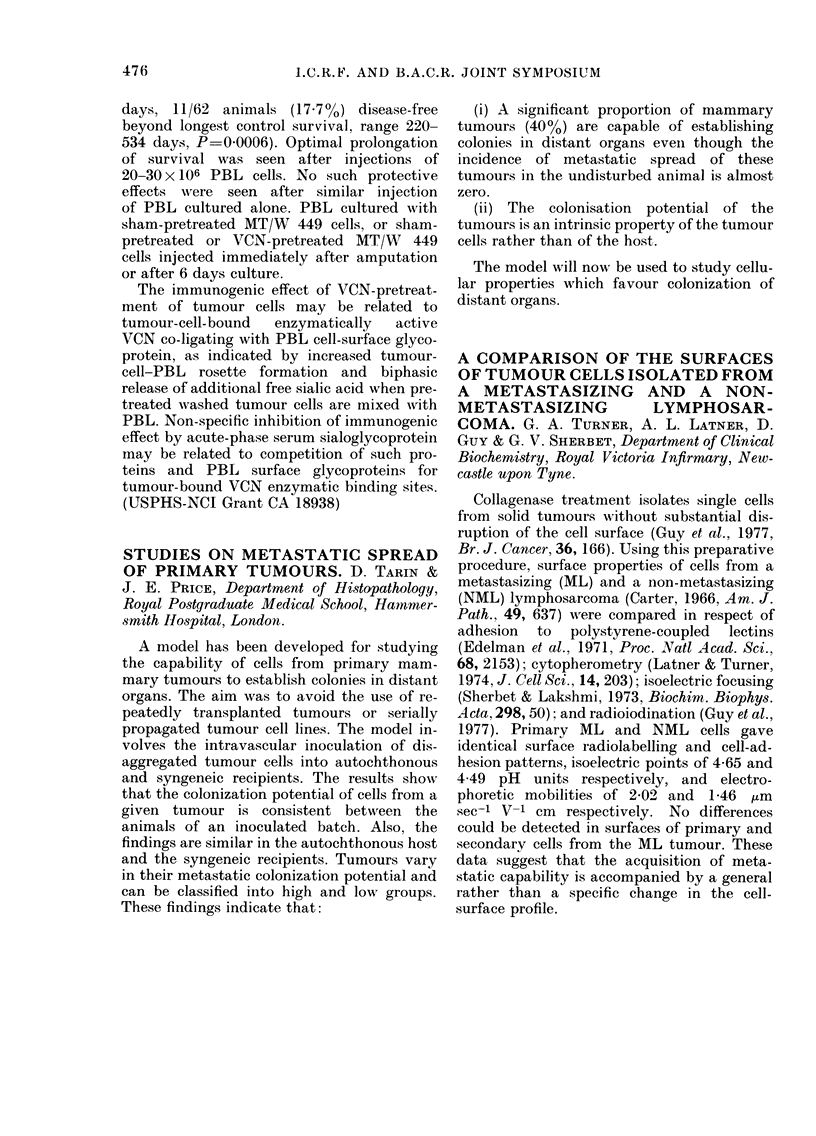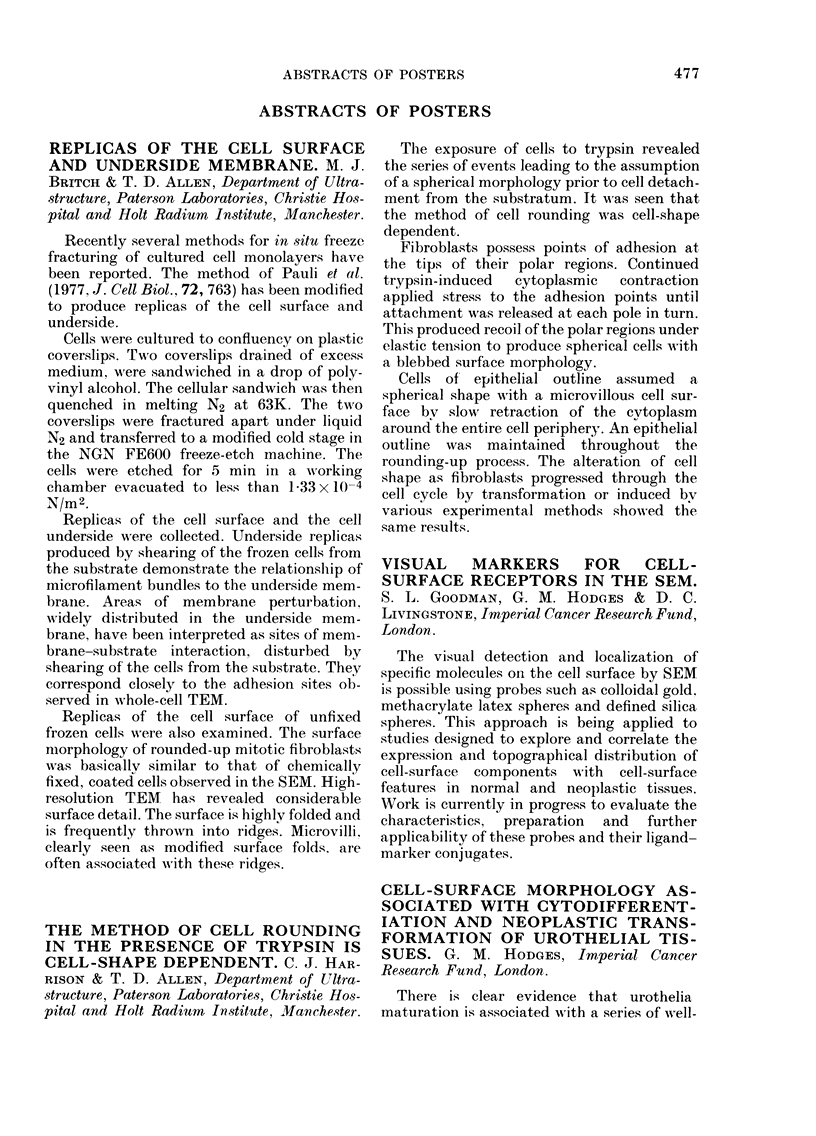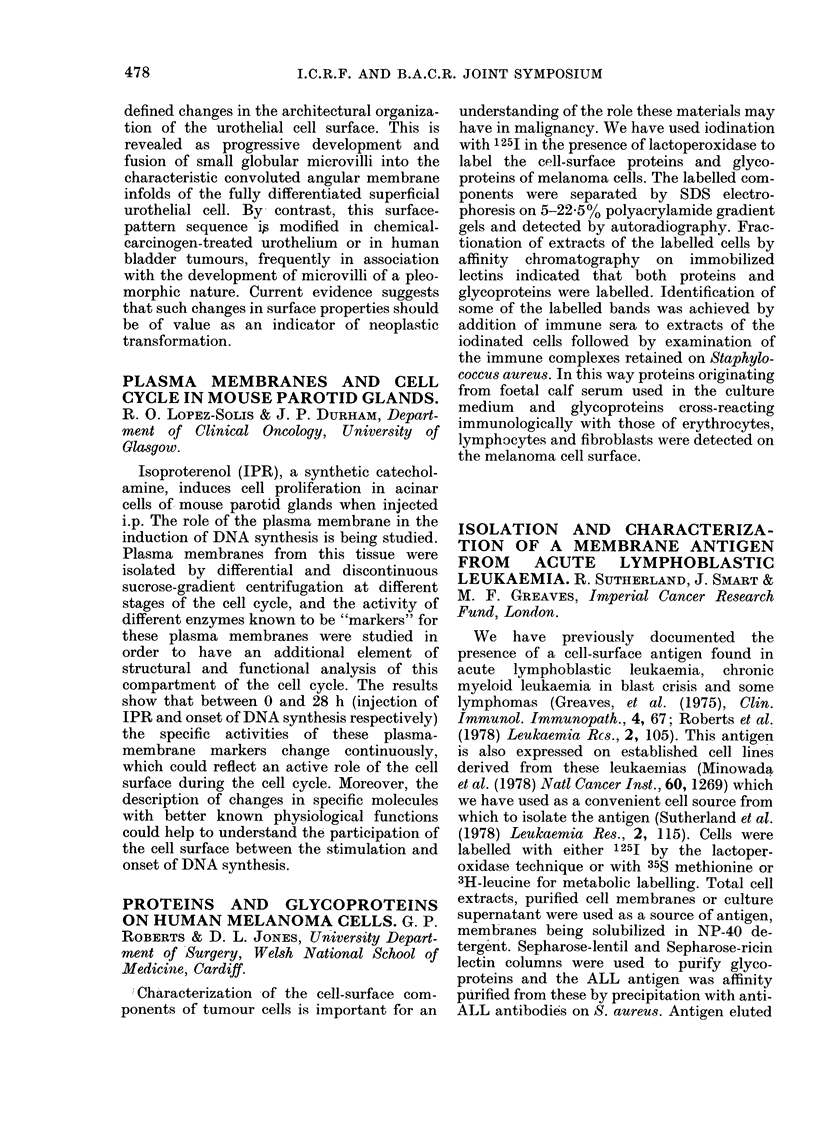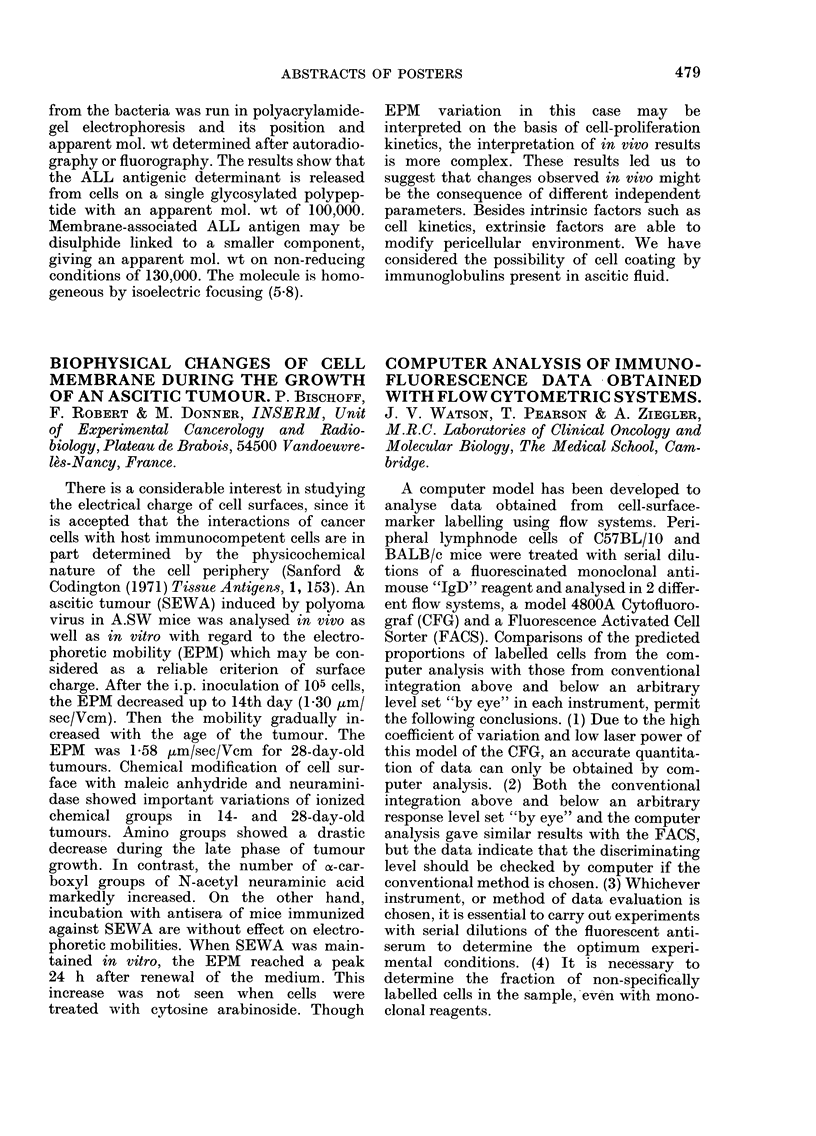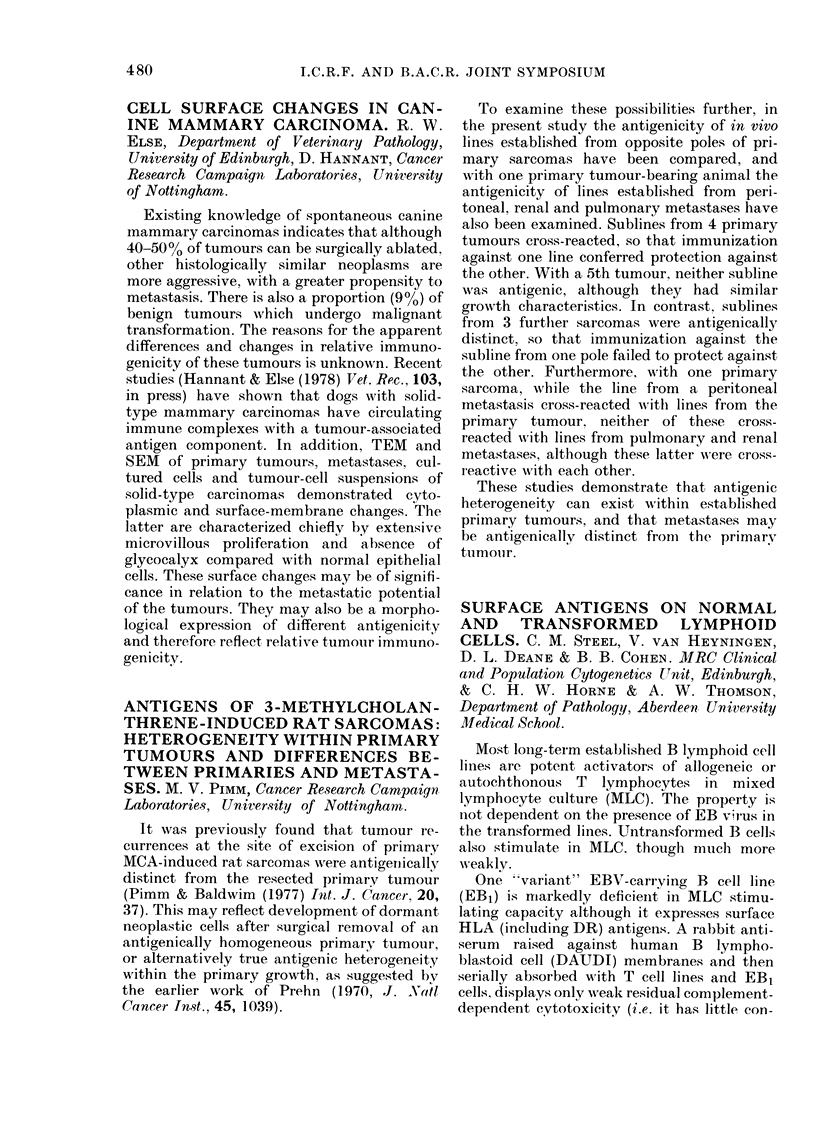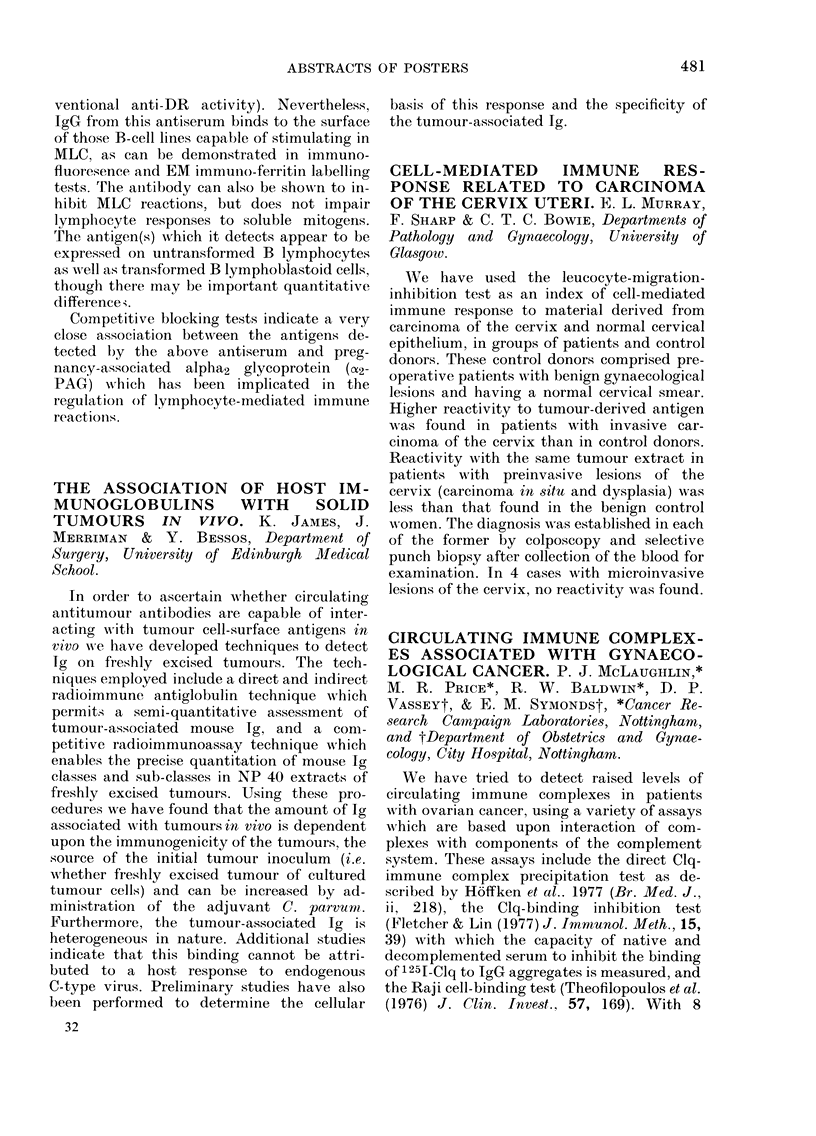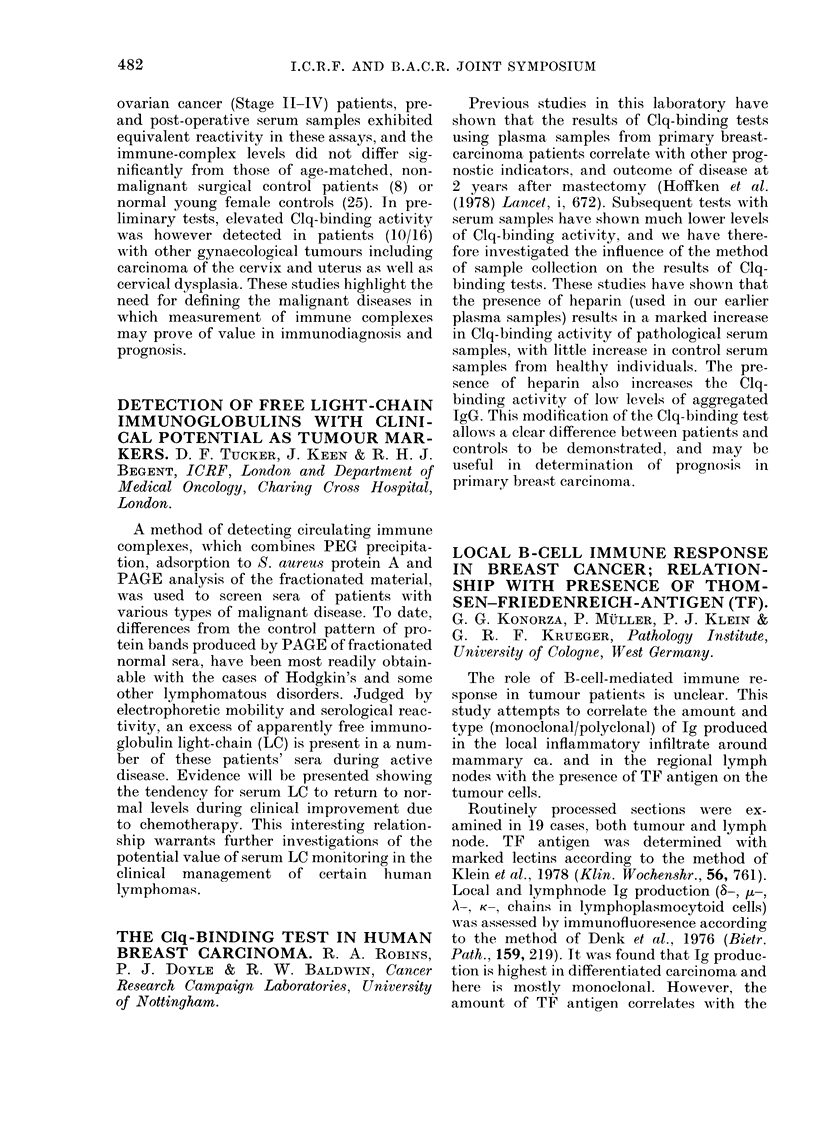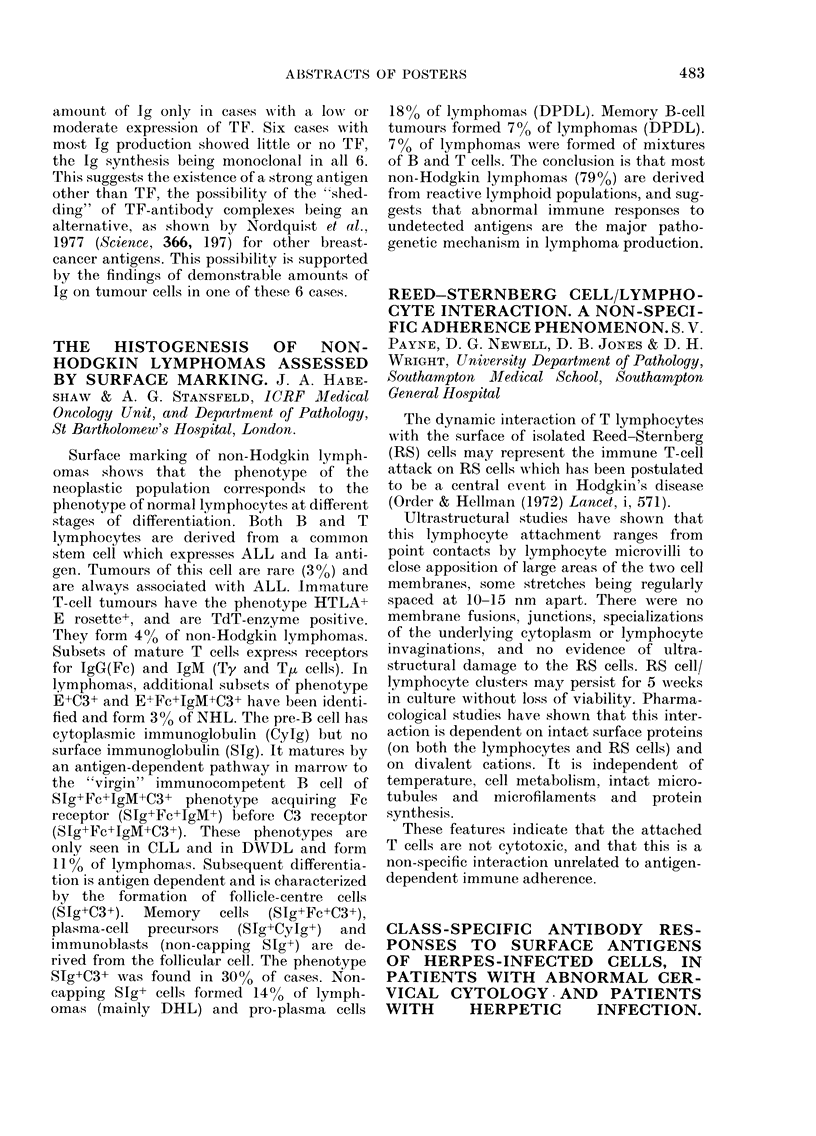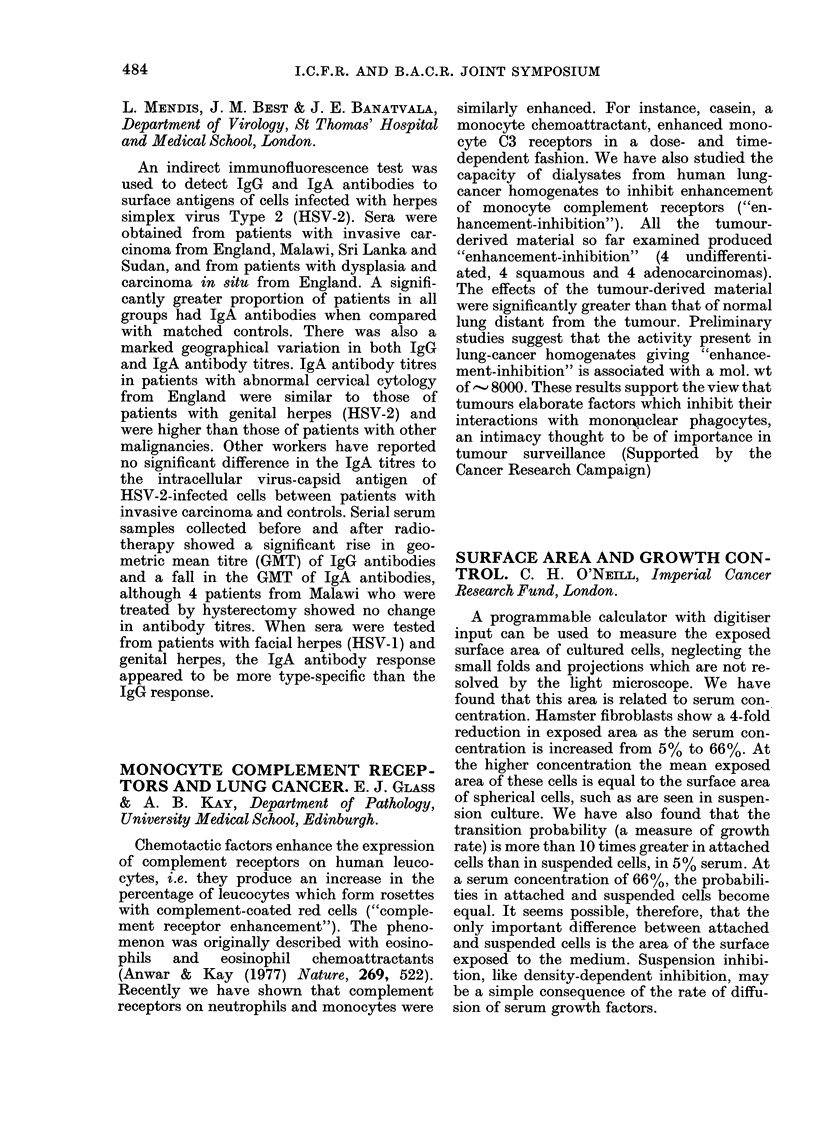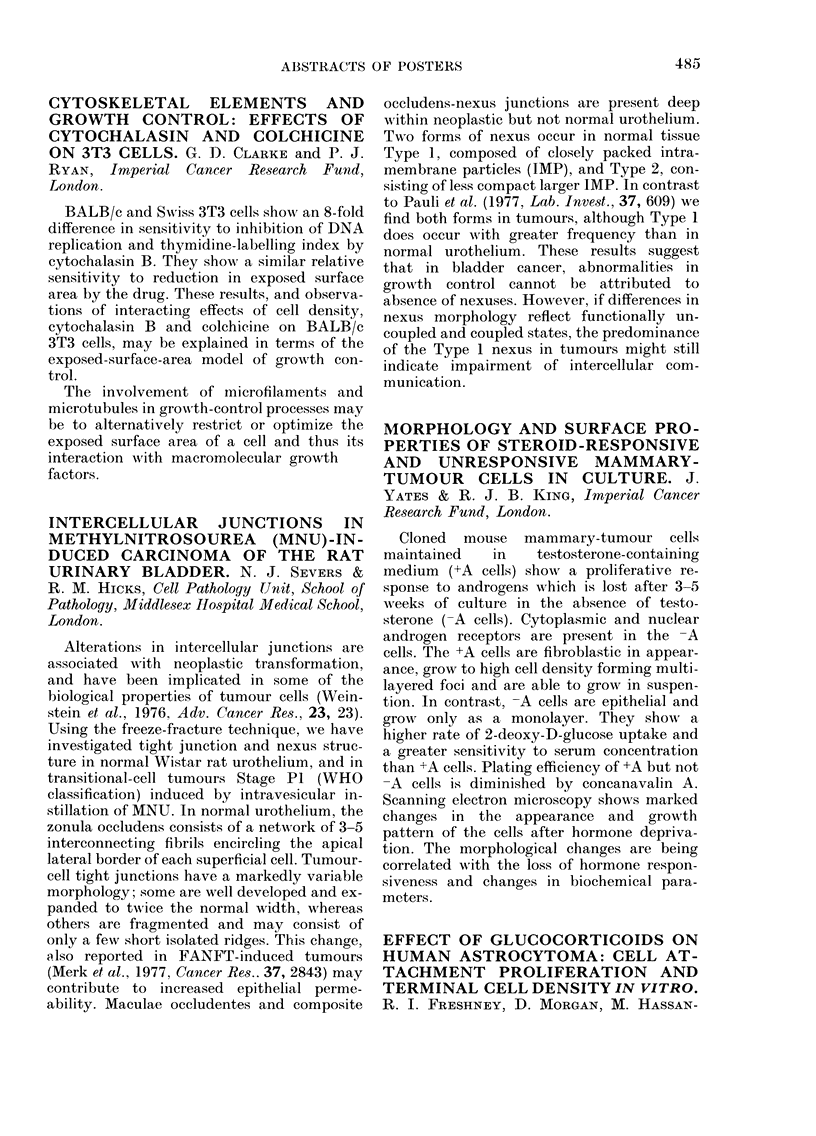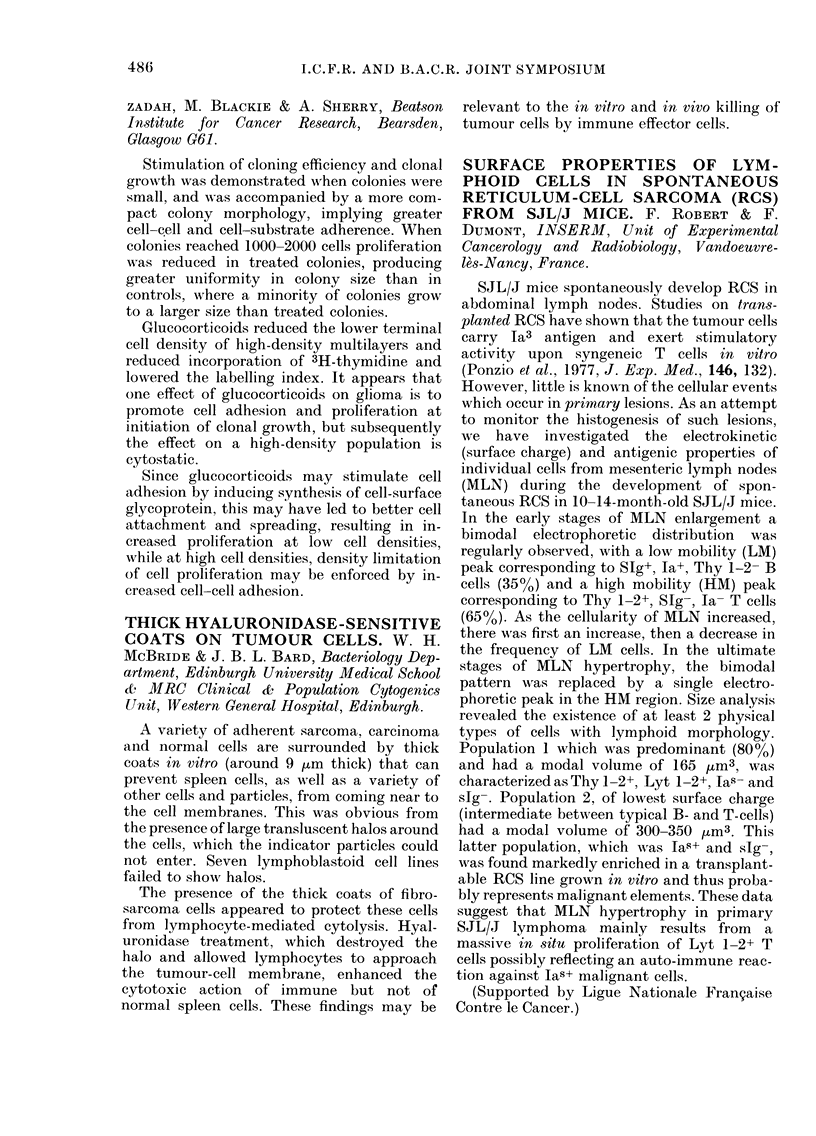# Imperial Cancer Research Fund British Association for Cancer Research. Joint symposium held at the Royal College of Surgeons, Lincolns Inn Fields, London 30 November--1 December 1978. Abstracts of papers.

**DOI:** 10.1038/bjc.1979.82

**Published:** 1979-04

**Authors:** 


*Br. J. Cancer* (1979) **39**, 457

### IMPERIAL CANCER RESEARCH FUND
### AND
### BRITISH ASSOCIATION FOR CANCER RESEARCH

### JOINT SYMPOSIUM

**Held at the Royal College of Surgeons, Lincolns Inn Fields,**

**London**

**30 November–1 December 1978**

### ABSTRACTS OF INVITED CONTRIBUTIONS

**GENETIC CONTROL OF CELL SURFACE COMPONENTS.** W. F. BODMER, *Genetics Laboratory, Department of Biochemistry, University of Oxford.*
***Title Only***

**VIRUS-ASSOCIATED CELL SURFACE PRODUCTS: RELEVANCE TO MALIGNANT TRANSFORMATION.** J. WYKE, *ICRF, London.*

Infection by viruses of several different families is associated with neoplasia, and many instances are known in which products associated with these viruses are situated at the surface of both normal and malignant cells. It has proved difficult to link these virus-specific cell-surface molecules with either the cause or the progression of malignancy. For this reason we will consider in basic terms the possible mechanisms of virus-induced transformation, and the role that virus-associated cell-surface components may play in such events, and we will attempt to assess the relevance of known virus-specific changes within this framework.

Cells may become transformed by viruses whose life cycle is lytic as well as by non-lytic agents. In the former case the cell survives, either because it is non-permissive or semi-permissive for virus replication or because virus replication is defective. In the latter instance similar constraints may reduce the level of virus expression, but it is also possible for the transformed cell to yield fully infectious virus. Whatever the nature of virus–cell interaction, the transformed cell always carries virus genetic material, and viruses could thus transform by interfering with normal cell controls in several ways. Firstly, integrated viral DNA may disrupt regulated expression of the cell genome, a situation which does not require the expression of viral genes. Secondly, virus-coded products may mimic the effect, or even the detailed action, of cell growth-promoting stimuli. If the virus-specific molecules are located in or near the cell membrane they may mimic exogenous signals acting across the cell surface, but the viral products may also resemble endogenous signals for growth.

Such virus-coded or virus-specific growth-promoting stimuli could be (1) structural proteins of the virus or their precursors (in their native form or modified by mutation, recombination or aberrant processing in an unusual host), (2) non-structural proteins involved in virus replication (which may also undergo modification), or (3) non-structural proteins with no apparent function in the virus life cycle. The latter class of potential transforming proteins may be particularly important in the Retroviridae, whose mode of replication and high rate of genetic recombination make it likely that the virus can acquire, by recombination, a new genetic material from either the host or another virus. The new material may comprise a clearly defined cistron, distinct from viral replicative functions, or it may be part of a hybrid gene (derived by recombination within a viral cistron) which codes for a protein with some amino acids in common with a virus protein and others of foreign origin. If retroviruses recombine with one another, the viral replicative functions could also be altered, and some oncogenic viruses seem to arise by recombination between two non-oncogenic parents.

Only two virus genes have been shown, by reasonable genetic and biochemical criteria, to be involved in causing cell transformation; the early region (T-antigen complex) of papovaviruses and the *src* gene of sarcoma-inducing retroviruses. It is possible that the proteins encoded in these genes may have similar mechanisms for inducing cell transformation but, whereas the papovavirus-coded proteins are involved in virus replication, the *src* gene product seems unrelated to the virus life cycle and this gene is possibly of host-cell origin. The cellular location of these proteins and their detailed mode of action is not understood, and it is possible that they may show functional polymorphism. One component of the T-antigen complex is membrane associated (though not known to be on the cell surface) and the expression of both the papovavirus and the retrovirus genes in the cell is closely correlated with the presence of virus-specific tumour antigens on the cell surface. However, the relationship between these antigens and the viral transforming genes is not understood.

Another group of retroviruses, typified by the erythroblastosis and myelocytoma viruses of chickens and the Abelson leukaemia virus of mice, are replication-defective agents which all encode a protein which comprises a portion of the retrovirus *gag* gene and amino acid sequences of an unknown origin. For some of these viruses there is preliminary genetic evidence that these hybrid proteins may be a cause of cell transformation, but their location in the infected cell is as yet unknown.

In the case of other leukaemia-inducing retroviruses there is no clear evidence for the involvement of a virus gene in the maintenance of the transformed state, but there is ample documentation of the presence of virus-associated antigens on the leukaemic cell surface. Some of these antigens, such as the mouse $G_{IX}$ thymocyte antigen and Gross cell-surface antigen (GCSA) were known to be associated with leukaemia-virus-induced neoplasms, but were thought possibly to be of non-viral origin. However, it is now known that $G_{IX}$ antigenicity is carried on the murine leukaemic virus (MuLV) envelope glycoprotein, whilst GCSA represents glycosylated forms of the precursors to the internal proteins of MuLV encoded by the viral *gag* gene. With increasing awareness of endogenous virus-specific proteins, including the *env* and *gag* gene products, in normal as well as transformed cells, it is difficult to assign to these antigens a function in the causation of leukaemia It is possible that they are simply evidence of viral gene activity, which may be a prerequisite for leukaemogenesis but which may also be apparent in other stages in the life of the host. However, though we do not know whether the virus antigens on the surface of the leukaemic cells have a causal role in the neoplasm, they, like the surface tumour antigens associated with sarcoma viruses, are invariably present. If this novel antigenicity elicits an immune response from the host, the virus-associated surface proteins may be an important factor in the progression of the disease.

### CELL-SURFACE PROPERTIES, GROWTH AND MALIGNANCY OF HAEMOPOIETIC CELL LINES.

K. Nilsson, *The Wallenberg Laboratory, University of Uppsala, Sweden.*

During the last few years, improved tissue-culture methodology has allowed the establishment of a number of cell lines *in vitro* from both normal and neoplastic human haemopoietic tissue and peripheral blood. Analyses of the phenotypic characteristics of haemopoietic lines have revealed that most of them are polyclonal and normal diploid in early passages, carry Epstein–Barr virus (EBV) and are therefore derived from presumably non-neoplastic precursor cells. Such cell lines, tentatively termed lymphoblastoid, can be established with ease from any anti-EBV seropositive individual. In contrast, truly neoplastic haemopoietic cell lines (leukaemia, lymphoma, myeloma) are still rare, and have been established only with great difficulty. The neoplastic lines are always monoclonal, aneuploid and, with exception for Burkitt's lymphoma lines, EBV-genome negative.

The lymphoblastoid lines (LCL) have a lymphoblastoid morphology, produce immunoglobulin (Ig) and express lymphocyte surface markers indicating a B-cell descent. The LCLs gradually evolve towards monoclonality and aneuploidy during continuous cultivation. This *in vitro* selection of an aneuploid clone usually occurs within one year of serial passage.

From the studies with the group of malignant haemopoietic lines a few general conclusions seem justified. Firstly, all the lines have individually distinct properties, suggesting that each human haemopoietic tumour, although sharing some basic morphological and functional features with other tumours belonging to the same histopathological entity, is unique. Secondly, haemopoietic tumours continue to express most of the features of the corresponding normal cells. Thirdly, the expression of the phenotypic properties is usually stable during at least 1–2 years of continuous cultivation *in vitro*. The leukaemic-cell lines are most frequently lymphoblastoid in morphology and usually have a T-cell-like phenotype. A few cell lines have surface characteristics indicating a non-T, non-B lymphocyte origin; even fewer lines have a suggestive B-cell origin. Recently, the first leukaemic cell line with myeloid characteristics has been established from a myeloid leukaemia. To date, 3 myeloma lines and 3 lymphocytic lymphoma lines have been reported. The myeloma lines express plasma-cell surface characteristics while the lymphocytic lymphoma lines have a B-cell phenotype. The largest number of lymphoma lines has been established from diffuse histiocytic lymphoma. The phenotypic variability among these lines is suggestive of a marked heterogeneity with respect to cell type of origin in tumours classified histologically as "histiocytic". The majority produce Ig, and thus seem to be derived from the B-lymphocyte series. Some lines have a lymphocyte morphology but express no lymphocyte surface markers, and have therefore tentatively been classified as being derived from non-T, non-B lymphocytes. Three lines have morphological, lymphocyte surface marker and enzyme characteristics, indicating descent from a monocyte type of cell, and would thus represent truly histiocytic lymphomas.

The aim of this overview is to describe some of our recent studies on cell-surface characteristics of malignant haemopoietic cell lines, with special reference to phenotypic properties assumed to be related to the neoplastic state of the cells. The main results will be briefly summarised in the following:

(1) Exposed surface glycoproteins (GP) have been studied in a panel of neoplastic (lymphoma, leukaemia, myeloma) and non-neoplastic (LCL) lines by the galactose oxidase-tritiated sodium borohydride labelling technique. The labelled GPs were separated by polyacrylamide slab-gel electrophoresis and visualized by autoradiography. Each type of malignant haemopoietic cell line was found to have a basic, easily distinguishable surface GP pattern. In addition, each line had one or a few unique GPs. The B and T lines had most GPs in common with normal B and T cells, respectively. The GPs most constantly associated with malignancy were found in B lymphomas as double bands with apparent mol. wts of 85–87 and 69–71. The nature of these GPs has not as yet been clarified. In leukaemia and myeloma lines, only unqiue GPs were found in addition to those shared with normal cells. In the group of histiocytic lymphoma lines 4 types of GP patterns could be distinguished: (a) a basically B-lymphocyte-like pattern in 2 lines expressing B-cell surface markers; (b) a basically T-lymphocyte-like pattern in 1 line with non-T, non-B surface phenotype; (c) a basically monocyte-like pattern in 1 line with a monocyte-like phenotype; (d) a "stem" cell-like pattern in 2 other lines with a histiocyte-like phenotype. The surface-labelling technique thus seems to be useful in discriminating the different types of "histiocytic" lymphomas.

(2) The fucose-labelled GPs of the haemopoietic lines were also characterized by gel filtration after degradation and release from the cell surface by proteolytic enzymes. The results demonstrate that the tumour lines, almost without exception, contain an increased amount of fast-eluting glycopeptides than a larger normal cell. Such fast-eluting glycopeptides have previously been demonstrated in several animals and human tumours, but not in normal cells. Interestingly, the surface glycopeptides of the LCL type of lines also had this "malignant" elution profile.

(3) The binding of the lectin *Helix pomatia* agglutinin (HP) to the surface of the various types of cell lines has been studied. In T lines a selective binding to a GP with an apparent mol. wt of 150 ku was found, whilst in B lymphoma and myeloma lines this lectin bound to a 210 GP. The non-T, non-B leukaemias bound HP to a 150 ku GP. The group of histiocytic lymphoma lines was again heterogeneous. The 2 $Ig^+$ lines expressed a 210 ku HP-binding surface GP, whilst the line with a non-T, non-B phenotype bound HP to a 150 ku GP. The 3 lines with a monocytic phenotype could be subdivided into 2 subgroups with respect to HP binding. The lysozyme-secreting U-937 line did not bind HP, whilst the 2 other lines had 3 HP-binding GPs of 210 ku, 150 ku and 70 ku. The HP studies confirm that the group of rare, truly histiocytic lymphomas also seems to be heterogeneous, and that such lymphomas may be derived from various stages of monocyte (histiocyte) differentiation.

(4) Employing a panel of antisera (NIH) to C-type virus-associated surface antigens in a screening by the staphylococcal protein A radioimmunoassay, most lines, including the many Burkitt's-lymphoma lines, were found to be negative. However, a few other lymphoma lines and one myeloma line reacted with anti-p30 and anti-GP70 ku.

(5) The tumorigenicity of the haemopoietic lines has been extensively studied by inoculation tests in nude mice. When inoculated s.c., most but not all neoplastic lines formed tumours, whilst the EBV-carrying diploid LCLs failed to grow. However, using the intracranial route, both the diploid LCL and the neoplastic lines grew. The implications of these results in relation to the putative oncogenic role of EBV will be discussed.

### GLYCOLIPIDS AND MALIGNANCY; A REVIEW OF SOME CURRENT IDEAS.

D. R. Critchley, *Department of Biochemistry, University of Leicester, Leicester.*

Experimental evidence has confirmed the hypothesis that changes in cell-surface organization are associated with the malignant cell.[1] One such change is loss of the more complex glycolipids from the malignant-cell surface, a consistent observation, whether the malignant cells are derived by viral or chemical transformation of stable cell lines or from spontaneously occurring or induced tumours (reference 2 for a review). The potential importance of the change has recently been emphasized by the demonstration that glycolipids *can* act as membrane receptors for a number of proteins, some of which in turn are able to influence intracellular events through elevation of cAMP levels. Current evidence suggests that several bacterial toxins (*e.g.* cholera[3,4] and tetanus toxins[5]), the glycoprotein hormones (thyrotropin (TSH),[6,7] luteinizing hormone (LH),[8] and human chorionic gonadotropin (HCG)[9]), and interferon[10,11] are all able to recognize specific carbohydrate sequences contained in glycolipids. Interestingly, the structure of cholera toxin has certain similarities with that of the glycoprotein hormones.[6,12]

Of the above examples, the interaction between cholera toxin and its putative receptor, glycolipid $GM_1$ (Table) has been the most extensively studied.

(a) Binding of toxin to target cells is abolished by pre-incubating the toxin with $GM_1$ (20 ng/ml), other glycolipids lacking the terminal galactose residue (*e.g.* $GM_2$, $GD_{1a}$ and $GM_3$) being much less effective inhibitors.[3]

(b) Cells lacking $GM_1$ do not bind or respond to cholera toxin, but can be made toxin responsive by incorporating exogenous $GM_1$ into their membrane.[13,14]

(c) Liposomes containing $GM_1$ bind cholera toxin.[15]

(d) Saturating levels of toxin protect the terminal galactose residue of $GM_1$ from galactose oxidase.[4,7]

(e) $GM_1$ (and the oligosaccharide derived from it) but not $GM_2$ or $GM_3$, causes a "blue-shift" in the fluorescence spectrum of the toxin, suggesting that the interaction leads to a conformational change in the toxin.[7,16]

We have recently extended these observations by showing that $GM_1$ is the only glycolipid recovered when intact surface-labelled (galactose oxidase or periodate/borotritiide[17]) BALB or Swiss 3T3 cells are exposed to toxin, and the toxin-receptor complexes isolated from NP40 extracts of the cells by addition of anti-toxin followed by formalin-treated *Staphylococcus aureus*.[18] Glycolipids $GM_3$ and $GD_{1a}$, the major cellular glycolipids and the ones most strongly labelled by the periodate procedure, were not recovered by this method (Critchley & Ansell, unpublished data).

Evidence that glycolipids are part of the glycoprotein hormone-receptor system stems largely from the following observations:

(a) Glycolipids inhibit hormone binding, each hormone having a distinctive glycolipid inhibition profile[6,8,9] (Table). A detailed examination of the glycolipids of the thyroid have resolved at least 28 sialic acid-containing glycolipids, one of which is markedly better at inhibiting TSH binding to thyroid membranes than the previous best inhibitor $GD_{1b}$.[19] The molecule, which has yet to be fully characterized, is a minor component of the total membrane glycolipid (0·015% of the total lipid-bound sialic acid; $\sim 10^4$ molecules per cell). Other glycolipids, *e.g.* minor components of testicular membranes, may well act as the *in vivo* receptors for HCG and LH.

TABLE—*The relative potencies of various glycolipids* in inhibiting toxin or hormone binding to target tissues.*

| | |
|---|---|
| CT | $GM_1 >> GD_{1a} > GM_2 > GT_1 >> GM_3$ (3) |
| Tetanus | $GD_{1b} = GT_1 > GM_1 > GD_{1a} > GM_2$ (5) |
| TSH | $GD1b > GT_1 > GM_1 > GM_2 = GM_3 > GD1a$ (6) |
| HCG | $GT_1 > GD_{1a} > GD_{1b} > GM_2 > GM_1$ (9) |
| LH | $GT_1 > GD1b >> GD1a > GM_1 > GM_2$ (8) |
| FSH | data not available |

*$GM_3$ = Cer-Glc-Gal-NAN;
$GM_2$ = Cer-Glc-Gal(NAN) GalNAc;
$GM_1$ = Cer-Glc-Gal(NAN)-GalNAc-Gal;
$GD_{1a}$ = Cer-Glc-Gal(NAN) GalNAc-Gal(NAN);
$GD_{1b}$ = Cer-Glc-Gal(NAN-NAN) GalNAc-Gal;
$GT_1$ = Cer-Glc-Gal(NAN-NAN) GalNAc-Gal(NAN).

Abbreviations: Cer, ceramide; Glc, glucose; Gal, galactose; GalNAc, N-acetylgalactosamine; NAN, N-acetylneuraminic acid.

(b) The change in the fluorescence spectrum of the hormone induced by the glycolipid which is the best inhibitor of binding is distinct from that produced by a minimal inhibitor of binding.[6,8,9]

(c) Also in support of the above data is the finding that the target tissues for TSH and HCG have unusually high levels of the more complex glycolipids for extraneural tissues.[6,8,9,19,20]

Initially these results appear surprising, in that glycoprotein receptors for TSH and

HCG have previously been isolated.[21,22] However, the primary determinant of the hormone–receptor interaction is probably a specific carbohydrate sequence, and it is not without precedent for the same carbohydrate sequence to appear in both glycolipid and glycoprotein.[23,24] Membranes from a thyroid tumour which is unresponsive to TSH not only lack the glycoprotein receptor, but levels of the more complex glycolipids are also markedly reduced.[25] In the light of the possible dual nature of the glycoprotein-hormone receptors, it is conceivable that a glycoprotein receptor for cholera toxin remains to be discovered.

However, some doubt about the role of glycolipids as the primary receptor for the glycoprotein hormones still remains, stemming from the observation that, whilst injection of HCG into rats leads to a reduction in HCG binding by testicular membranes, the glycolipid profile of the membranes is unaltered.[20] In addition, glycolipids extracted from the testes of treated rats were as effective as those from control animals in inhibiting $^{125}$I-HCG binding by isolated testicular membranes. Similar questions are posed by the finding that whilst thyroid cells from patients with Grave's disease bind normal amounts of TSH, the glycolipid profile of the cells is altered.[26] It would be interesting to re-examine the glycolipid profile of these cells in the light of the more recent data showing that the putative glycolipid receptor for TSH is a very minor component of the thyroid cell membrane.[19]

Given that glycolipids are candidate membrane receptors for certain hormones, their loss from the tumour-cell surface may well place the cells outside the sphere of influence of those mechanisms which normally regulate the expression of certain of the differentiated cellular functions. An additional role for glycolipids in cell–cell-interaction has been proposed.[27] Recent evidence suggests that glycolipids are important cell-surface determinants in retinotectal adhesion[28] and nerve-muscle interaction.[29] Clearly, were these observations confirmed and extended, loss of cell-surface glycolipids might be an important factor in the breakdown in cellular interaction characteristic of malignant cells.[2]

REFERENCES

1 Nicholson, G. L. (1970) *Biochim. Biophys. Acta*, **458**, 1.

2 Critchley, D. R. & Vicker, M. G. (1977) In *Cell Surface Reviews.*

3 Cuatrecasas, P. (1973) *Biochemistry*, **12**, 3558.

4 Moss, J., Manganiello, V. C. & Fishman, P. H. (1977) *Biochemistry*, **16**, 1876.

5 Ledley, F. D., Lee, G., Kohn, L. D., Habig, W. H. & Hardegree, M. C. (1977) *J. Biol. Chem.*, **252**, 4049.

6 Mullin, B. R., Fishman, P. H., Lee, G. & 5 others (1976) *Proc. Natl Acad. Sci. U.S.A.*, **73**, 842.

7 Mullin, B. R., Aloj, S. M., Fishman, P. H., Lee, G., Kohn, L. D. & Brady, R. O. (1976) *Proc. Natl Acad. Sci. U.S.A.*, **73**, 1679.

8 Lee, G., Aloj, S. M. & Kohn, L. D. (1977) *Biochem. Biophys. Res. Commun.*, **77**, 434.

9 Lee, G., Aloj, S. M., Brady, R. O. & Kohn, L. D. (1976) *Biochem. Biophys. Res. Commun.*, **73**, 370.

10 Besancon, F., Anlek, H. & Basu, S. (1976) *Nature*, **259**, 576.

11 Vengris, V. E., Reynolds, F. H., Hollenberg, M. D. & Pitha, P. M. (1976) *Virology*, **72**, 486.

12 Kurosky, A., Markel, D. E. & Fitch, W. M. (1977) *Science*, **195**, 299.

13 Moss, J., Fishman, P. H., Manganiello, V. C., Vaughan, M. & Brady, R. O. (1976) *Proc. Natl Acad. Sci. U.S.A.*, **73**, 1034.

14 Fishman, P. H., Moss, J. & Vaughan, M. (1976) *J. Biol. Chem.*, **251**, 4490.

15 Moss, J., Fishman, P. H., Richard, R. L., Alving, C. R., Vaughan, M. & Brady, R. O. (1976) *Proc. Natl Acad. Sci. U.S.A.*, **73**, 3480.

16 Fishman, P. H. Moss, J. & Osborne, J. C. (1978) *Biochemistry*, **17**, 711.

17 Moss, J., Manganiello, V. C. & Fishman, P. H. (1977) *Biochemistry*, **16**, 1876.

18 Kessler, S. W. (1975) *J. Immunol.*, **115**, 1617.

19 Mullin, B. R., Pacuzka, T., Lee, G., Kohn, L. D., Brady, R. O. & Fishman, P. H. (1977) *Science*, **199**, 77.

20 Pacuska, T., Osborne, J. C., Brady, R. O. & Fishman, P. H. (1978) *Proc. Natl Acad. Sci. U.S.A.*, **75**, 764.

21 Tate, R. L., Holmes, J. M., Kohn, L. D. & Winard, R. J. (1975) *J. Biol. Chem.*, **250**, 6527.

22 Dufau, M. I., Ryan, D. W., Baukal, A. J. & Catt, K. J. (1975) *J. Biol. Chem.*, **250**, 4822.

23 Watkins, W. M. (1976) *Science*, **152**, 172.

24 Tonegawa, Y. & Hakamori, S. (1977) *Biochem. Biophys. Res. Commun.*, **76**, 9.

25 Meldolesi, M. F., Fishman, P. H. Aloj, S. M., Kohn, L. D. & Brady, R. O. (1976) *Proc. Natl Acad. Sci. U.S.A.*, **73**, 4060.

26 Lee, G., Grollman, E. F., Aloj, S. M., Kohn, L. D. & Winard, R. J. (1977) *Biochem. Biophys. Res. Commun.*, **77**, 139.

27 Hakamori, S. (1975) *Biochim. Biophys. Acta*, **417**, 53.

28 Marchase, R. B. (1977) *J. Cell Biol.*, **75**, 237.

29 Obata, K., Oide, M. & Handa, S. (1977) *Nature*, **266**, 369.

### CELL-SURFACE PROTEINS AND THE TRANSFORMED PHENOTYPE. R. Hynes, *Dept. of Biology and Center for Cancer Research, M.I.T., Cambridge, Mass., U.S.A.*

A variety of phenotypic properties of oncogenically transformed cells involve cellsurface proteins. Thus, cellular growth control may operate through cell–cell contact or through the binding of hormones or growth factors to cell-surface receptors. Growth control is altered by transformation and, whichever control mechanism operates is likely to involve surface proteins. Cellular adhesion and a complex of related properties (morphology, cytoskeleton, migration, contact inhibition of movement) are also frequently altered by malignant transformation. Finally, tumour cells bear neoantigens against which immune responses are mounted. For all these reasons it is reasonable to inquire into alterations in cell-surface proteins.

During the past several years a number of alterations in surface and membrane proteins have been described.[1] The challenge is to determine which of these changes, if any, are involved in the altered phenotype of transformed cells.

One protein has been particularly extensively studied. It is a large glycoprotein of subunit mol. wt 210–250 ku and is commonly known as LETS protein or fibronectin. Fibronectin is a major surface protein of fibroblasts, and also occurs on some other cell types (myoblasts, endothelial cells, amniotic cells, some epithelial cells in culture, and maybe others). It is not a typical membrane protein and depending on one's point of view it can be considered as a peripheral or extrinsic membrane protein, as a constituent of the cell-surface coat, or as part of the extracellular matrix. A fraction rich in fibronectin but containing little or no lipid can be separated from plasma membranes.[4,5] This fraction appears to be the cell-surface coat and contains a variety of other glycoproteins. Immunofluorescence studies show that fibronectin occurs in fibrillar networks under, over and between cells.[6–9] The exact pattern depends on the cell type and the culture conditions.

The properties of cell-surface fibronectin (LETS protein) which make it of interest in the context of oncogenic transformation are that it is regulated in amount in normal cells, depending on their density and state of growth[10], and that it is absent or greatly reduced in most transformed cells. The correlation between transformation or tumorigenicity and loss of fibronectin is good, but not perfect.[1,3,12,13,] Exceptions can be found. However, in cells transformed by tumour viruses temperature-sensitive for transformation, loss of fibronectin is also temperature-sensitive as expected for a property closely linked with the transformed phenotype.

The reasons for the reduced levels of fibronectin on transformed cells are not yet fully understood. Part of the explanation lies in a reduced level of synthesis, but this is not a sufficient explanation.[17,18] Normal cells also regulate their rate of biosynthesis, growing cells synthesizing fibronectin at a lower rate than growth-arrested ones[19]. This appears to be a sufficient explanation for the reduced levels on normal growing cells and may be the reason for the reduced rate of synthesis in transformed cells which are, naturally, growing. However, there is a depression in surface levels over and above this effect. Transformed cells also bind fibronectin less well than normal cells.[19,20] Attempts to explain the loss of fibronectin, which is extremely sensitive to proteases, as a consequence of activation of plasminogen by plasminogen activator produced by transformed cells, have led to the conclusions that plasminogen activation is neither necessary nor sufficient, and that proteolysis of fibronectin is an unlikely explanation for its absence.[13,21–3]: It remains possible that some other molecule to which fibronectin binds is either absent in transformed cells or is degraded by proteolysis.

To determine which aspects of the transformed phenotype might be related to the absence of fibronectin, the protein can be purified from normal cells and added to transformed cultures.[24,25] The added fibronectin binds to the transformed cells in a fibrillar network very similar to that on normal cells.[25] The transformed cells show increased cell–substratum attachment and spreading, and reduced cell overlapping. With increasing doses, the cells become elongated and aligned like normal fibroblasts. All of these effects can be viewed as consequences of increased adhesion mediated by the added fibronectin. It now appears that contact inhibition of movement is a consequence of adhesion between cells and substratum, which is reduced in transformed cells.[26] Thus, one can hypothesize that addition of fibronectin produces increased adhesion and decreased cell over- or underlapping, leading to contact inhibition of movement and cell alignment. The corollary of this hypothesis is that reduced levels of fibronectin in transformed cells lead to reduced adhesion, and that several other parameters of the transformed phenotype

(rounded morphology, surface ruffles, loss of contact, inhibition of movement, overlapping and multilayering) follow from this. It has also been shown that exogenously added fibronectin increases cell spreading and migration in normal cells.[27] These results conform with the observation by immunofluorescence microscopy that fibronectin is arrayed in characteristic fibrillar patterns beneath the spreading lamellae of spreading or migrating cells.[19]

Another parameter of transformation which may or may not be related to cellular adhesion and morphology is the apparent disorganization of the microfilamentous arrays in transformed cells. Normal cells possess actin microfilaments arrayed in submembranous sheaths and in bundles traversing the cell. Electron and immunofluorescent microscopy have shown that these arrays are less well-developed in most transformed cells. It is of some interest that, when fibronectin is added to transformed cells, the microfilament bundles reappear.[25,28] Conversely, when the microfilament bundles of normal cells are disrupted by cytochalasin B, fibronectin is released from the cell surface.[29] These results suggest that fibronectin and actin might be connected. Double-label immunofluorescence shows that, indeed, the two arrays of filaments show strong correspondences under a variety of conditions.[30]

It is conceivable, therefore, that fibronectin is involved in adhesion and, in particular, in the formation of adhesion plaques to which actin microfilament bundles are attached, and that this involvement is the basis for its participation in a variety of cellular properties which alter on oncogenic transformation.

REFERENCES

[1] Hynes, R. O. (1976) *Biochim. Biophys. Acta*, **458**, 73.
[2] Yamada, K. M., & Olden, K. (1978) *Nature*, **275**, 179.
[3] Vaheri, A. & Mosher, D. (1978) *Biochim. Biophys. Acta*, **516**, 1.
[4] Graham, J. M., Hynes, R. O., Davidson, E. A. & Bainton, D. F. (1975) *Cell*, **4**, 353.
[5] Graham, J. M., Hynes, R. O., Rowlatt, C. & Sandall, J. K. (1978) *Ann. N.Y. Acad. Sci.*, **312**, 221.
[6] Mautner, V. & Hynes, R. O. (1977) *J. Cell Biol.*, **75**, 743.
[7] Hedman, K., Vaheri, A. & Wartiovaara, J. (1978) *J. Cell Biol.*, **76**, 748.
[8] Chen, L. B., Murray, A., Segal, R. A., Bushnell, A. & Walsh, M. L. (1978) *Cell*, **14**, 377.
[9] Yamada, K. M. (1978) *J. Cell Biol.*, **78**, 520.
[10] Hynes, R. O. & Bye, J. M. (1974) *Cell*, **3**, 113.
[11] Hynes, R. O. (1973) *Proc. Natl Acad. Sci. U.S.A.*, **70**, 3170.
[12] Chen, L. B., Gallimore, P. H. & McDougall, J. K. (1976) *Proc. Natl Acad. Sci. U.S.A.*, **73**, 3570.
[13] Pearlstein, E., Hynes, R. O., Franks, L. M. & Hemmings, V. J. (1976) *Cancer Res.*, **36**, 1475.
[14] Robbins, P. W., Wickus, G. G., Branton, P. E. & 4 others (1974) *Cold Spring Harbor Symp.*, **39**, 1173.
[15] Hynes, R. O. & Wyke, J. A. (1975) *Virology*, **64**, 492.
[16] Vaheri, A. & Ruoslahti, E. (1974) *Int. J. Cancer* **13**, 579.
[17] Olden, K. & Yamada, K. M. (1977) *Cell*, **11**, 957.
[18] Hynes, R. O., Destree, A. T., Mautner, V. & Ali, I. U. (1977) *J. Supramol. Struct.*, **7**, 397.
[19] Hynes, R. O., Ali, I. U., Destree, A. T. & 5 others (1978) *Ann. N.Y. Acad. Sci.*, **312**, 317.
[20] Perkins, M. E., Ji, T. H. & Hynes, R. O. (1979) submitted for publication.
[21] Hynes, R. O., Wyke, J. A., Bye, J. M., Humphreys, K. C. & Pearlstein, E. S. (1975) *Proteases and Biological Control*, p. 931.
[22] Hynes, R. O. & Pearlstein, E. S. (1976) *J. Supramol. Struct.*, **4**, 1.
[23] Mahdavi, V. & Hynes, R. O. (1978) *Biochim. Biophys. Acta*, **542**, 191.
[24] Yamada, M. K., Yamada, S. S., & Pastan, I. (1976) *Proc. Natl Acad. Sci. U.S.A.*, **73**, 1217.
[25] Ali, I. U., Mautner, V., Lanza, R. & Hynes, R. O. (1977) *Cell*, **11**, 115.
[26] Bell, P. B. (1977) *J. Cell Biol.*, **74**, 963.
[27] Ali, I. U. & Hynes, R. O. (1978) *Cell*, **14**, 439.
[28] Willingham, M. C., Yamada, K. M., Yamada, S. S., Pouyssegur, J. & Pastan, I. (1977) *Cell*, **10**, 375.
[29] Ali, I. U. & Hynes, R. O. (1977) *Biochim. Biophys. Acta*, **471**, 16.
[30] Hynes, R. O. & Destree, A. T. (1978) *Cell*, **15**, 875.

### THE ACTION OF GROWTH FACTORS IN NORMAL AND TRANSFORMED FIBROBLASTS IN CULTURE.

P. S. Rudland* & L. Jimenez de Asua†, **Imperial Cancer Research Fund, London, and †Friedrich Miescher-Institut, Basel.*

Hormones are known to control many processes of cell replication as well as those of metabolic activity in the animal. However, their effects in promoting proliferation of fibroblastic cells in tissue culture are small compared with those obtainable with whole serum. This suggests that the effects of whole serum are produced by other agents and there is evidence that low molecular proteins may be important amongst these. Here we shall confine ourselves to discussing growth factors which affect cultured fibroblastic cells, since little detailed work has been done in other systems. We shall define a growth factor for a given cell as a component which will stimulate the multiplication of cells in a nutritionally and otherwise complete medium for that cell, at concentrations which are considered to be near its physiological level.[2] Growth factors are probably best classified according to their source, since relatively little is known about their site of action in the animal. We shall deal mainly with the pure polypeptides, pituitary fibroblast growth factor, FGF,[3] submaxillary epidermal growth factor, EGF,[4] and the prostaglandin $F_{2}\alpha$, $PGF_{2}\alpha$[5] produced by many cells. Most of the growth factors have their growth-promoting activity modified by certain metabolic hormones such as hydrocortisone and insulin, which alone have little or no effect on cell proliferation.[2]

Before considering possible biochemical mechanisms for the action of growth factors, we should describe the observable effect of growth factors on the cell cycle. When FGF and hydrocortisone or 10% serum are added to confluent, slowly proliferating BALB/c 3T3 cells (quiescent cultures) grown in medium containing serum, the kinetics for the increase in the fraction of cells which have entered S phase are nearly identical to the kinetics for the increase in the number of cells, except that the latter increase is delayed by 9–10 h.[6] The simplest explanation of this is that the duration of the remainder of the cell cycle up to division is constant.[6] In another system (3T6 cells) when serum is completely removed from the medium the cells require additional components to progress through later stages of the cell cycle, but these additional components probably do not have a regulatory role.[7] Growth factors such as FGF,[8] and $PGF_{2}\alpha$[9] when added to quiescent 3T3 cells cause an abrupt increase in the rate of cellular entry into S phase after a constant lag period. During the lag period cells are entering S phase at a very low rate. The initial and final kinetics seem to be first order for $PGF_{2}\alpha$[9] as they are for serum,[10] and can therefore be described by a first-order rate constant $k$; increasing concentrations of $PGF_{2}\alpha$ increase $k$. This observation is consistent with the model of the cell cycle for cells growing under constant conditions proposed by Burns and Tannock[11] and Smith and Martin[12] in which a random decay from part of the $G_1$ phase to the remainder of the cell cycle is governed by a first-order rate constant $\gamma$ or $k_{trans}$. To explain the kinetics obtained by adding $PGF_{2}\alpha$ or FGF at different concentrations and different times in the lag phase, two signals, *1* and *2* are postulated. Signal *1* initiates the lag phase and signal *2* determines the value of $k$. The times during the lag phase when hormones must be present to effect changes in the value of $k$ suggest that the pathway triggered by signal *1* is organised in a linear sequence of steps during this period, and that the change in the putative rate-limiting step which is responsible for first-order kinetics is situated near the end of the lag phase.[9,13,14] Similar conclusions concerning the position of this "rate-limiting step" have been obtained from experiments with inhibitors of protein synthesis.[15]

Turning to the detailed study of the interaction of growth factors with their target cells, two basic mechanisms have been suggested for EGF. (1) The growth factor binds to specific receptors in the plasma membrane[16] and thereby triggers specific messengers[1] (cyclic nucleotides, ions, nutrients) which continue the stimulus inside the cell. (2) The growth factor is internalized and goes directly to intracellular sites[17] concerned with the growth stimulus. These possible effects may not be mutually exclusive. Three lines of evidence suggest that the first mechanism cannot be the complete story. (1) 3T3 cells partially starved for certain nutrients cannot increase their proliferation rates with FGF.[18] (2) The magnitude of early changes[1,19] in cyclic nucleotide and ion concentrations and transport of nutrients triggered by $PGF_{2}\alpha$ or FGF do not necessarily show correlations with the final value of $k$.[2,14] (3) EGF stimulates many of the early changes in a 3T3 cell variant, but fails to increase the value of $k$, whereas $PGF_{2}\alpha$ behaves normally, suggesting an EGF-specific interactior with the cell beyond plasma-membrane binding (unpublished results). On the other hand there is no evidence as yet to suggest that internalization and accumulation of EGF by the cell is involved in the delivery of the mitogenic signal.[20]

Even more difficult to understand is the constancy of the lag period, independent of PGF or $PGF_{2}\alpha$ concentrations, before increased values of $k$ are observed. We have suggested[2] that the constancy of this period is due to a requirement for interaction of the pathways generated by signal *1* and signal *2* before an intracellular component becomes "competent" to cause increased values of $k$ at the end of the lag. The nature of this hypothetical component is unknown, but since only the later events which result from changes in the amount of proteins inside the cell show consistent correlations with the value of $k$ to date,[2] and since there is a strict requirement for protein synthesis for changes in $k$ at the end of the lag phase,[15] it is not unreasonable to suppose either that this component may be a protein which has to be modified or a modification to the cell has to take place before this component becomes "competent" to cause an increase in $k$. One obvious candidate is a cellular equivalent to the small DNA tumour-virus nuclear T antigen which can stimulate host-cell DNA synthesis when injected directly into quiescent cells.[21] Interestingly, the apparent relative rate of accumulation of a nuclear non-histone protein seen near the end of the lag phase after addition of $PGF_{2\alpha}$ to quiescent cells is correlated with the final value of $k$.[22] Experiments are in progress to isolate this and other proteins, and to test for their effect on $k$ by direct microinjection into quiescent cells (L. J. de A., unpublished).

Chemical or viral transformation of fibroblasts in tissue culture produces cell variants with different properties from the original cells, including increased ability to form tumours in animals (review ref. 23). One different property is their response to growth factors. Thus FGF can stimulate cell replication in untransformed 3T3 cells, but not in polyoma or SV 40-transformed 3T3 cells[6] or in 3T3 cell transformation mutants at the temperature at which the cells are transformed.[24] It has been suggested that a possible mechanism for transformation *in vitro* and formation of a neoplastic cell *in vivo* is due to an increased ability of cells to produce their own growth factors, so ensuring a degree of cellular autonomy from external control by growth factors present in serum.[25,26] In particular, the fact that murine sarcoma virus-transformed fibroblasts fail to bind EGF at the cell's surface but produce EGF-like polypeptides which compete for binding with EGF to cultured cells, has suggested that the transforming *sarc* gene product may be a growth factor which binds to the plasma membrane, causing the altered properties and failure of these cells to bind EGF.[27] This hypothesis fails to explain some of the other changes which accompany transformation in fibroblastic cells (*e.g.* cell-surface glycoprotein changes, increased protease activity, reduced cell-adhesive properties) which can be observed independently of changes in proliferation rates in temperature-sensitive mutant BALB/c 3T3 cells.[28,29] A more likely explanation is that the *sarc* gene product has more general effects on the plasma membrane, one of which may be to alter its binding of certain growth factors. However, it may not be unreasonable to conceive that one of the transforming viral gene products associated with increasing host-cell proliferation rates, the nuclear T antigen of the small DNA tumour viruses, may be acting like the hypothetical host-cell "component" for maintaining increased cellular proliferation rates. In this way the transformed cell may perpetuate the stimulus for increased values of $k$ that the growth factor produces in untransformed cells.

### REFERENCES

1 Holley, R. W. (1975) *Nature*, **258**, 487.
2 Rudland, P. S. & Jimenez de Asua, L. (1978) *Biochem. Biophys. Acta Cancer Rev.* (in press).
3 Gospodarowicz, D. (1975) *J. Biol. Chem.*, **250**, 2515.
4 Cohen, S., Taylor, J. M. & Savage, C. R. (1974) *Rec. Prog. in Hormone Res.*, **30**, 533.
5 Jimenez de Asua, L., Clingan, D. & Rudland, P. S. *Proc. Natl Acad. Sci. U.S.A.*, **72**, 2274.
6 Rudland, P. S., Seifert, W. & Gospodarowicz, D. (1974) *Proc. Natl Acad. Sci. U.S.A.*, **71**, 2600.
7 Rudland, P. S., Durbin, H., Clingan, D., & Jimenez de Asua, L. (1977) *Biochem. Biophys. Res. Comm.*, **75**, 556.
8 Mierzejewski, K., & Rozengurt, E. (1976) *Biochem. Biophys. Res. Comm.*, **73**, 271.
9 Jimenez de Asua, L., O'Farrell, M. K., Bennett, D. C., Clingan, D. & Rudland, P. S. (1977) *Nature*, **265**, 151.
10 Brooks, R. (1975) *J. Cell Physiol.*, **86**, 369.
11 Burns, F. J. & Tannock, J. F. (1970) *Cell Tissue Kinetics*, **3**, 321.
12 Smith, J. A. & Martin, L. (1973) *Proc. Natl Acad. Sci. U.S.A.*, **70**, 1263.
13 Jimenez de Asua, L., O'Farrell, M. K., Clingan, D. & Rudland, P. S. (1977) *Proc. Natl Acad. Sci. U.S.A.*. **74**, 3845.
14 Jimenez de Asua, L., Richmond, K. M., O'Farrell, M. K., Otto, A. M., Kubler, A. M. & Rudland, P. S. (1978) *Cold Spr. Hb Symp. Hormones and Cell Culture* (in press).
15 Brooks, R. (1977) *Cell*, **12**, 311.
16 Hollenberg, M. D. & Cuatrecasas, P. (1975) *J. Biol. Chem.*, **250**, 3845.
17 Carpenter, G., & Cohen, S. (1977) *J. Cell Biol.*, **250**, 3845.
18 Kamely, D. & Rudland, P. S. (1976) *Nature*, **260**, 51.
19 Rozengurt, E. (1976) *J. Cell Physiol.*, **89**, 627.
20 *Cold Spr. Hb Symp. Hormones and Cell Culture* (1978) (in press).
21 Tjian, R., Fey, G. & Graessmann, A. (1978) *Proc. Natl Acad. Sci. U.S.A.*, **75**, 1279.

22 O'FARRELL, M. & DIXON, C. (manuscript in preparation).
23 PASTAN, I. & WILLINGHAM, M. (1978) *Nature*, **274**, 645.
24 RUDLAND, P. S., ECKHART, W., GOSPODAROWICZ, D. & SEIFERT, W. E. (1974) *Nature*, **250**, 337.
25 BURK, R. R. (1973) *Proc. Natl Acad. Sci. U.S.A.*, **70**, 369.
26 TODARO, G. J., DELARCO, J. E. & COHEN, S. (1976) *Nature*, **264**, 26.
27 DELARCO, J. E. & TODARO, G. J. (1978) *Proc. Natl Acad. Sci. U.S.A.*, **75**, 4001.
28 RUDLAND, P. S., PEARLSTEIN, E., KAMELY, D., NUTT, M. & ECKHART, W. (1975) *Nature*, **256**, 43.
29 POUYSSEGUR, J. M. & PASTAN, I. (1976) *Proc. Natl Acad. Sci. U.S.A.*, **73**, 544.

### CELL COMMUNICATION IN NORMAL AND MALIGNANT CELL POPULATIONS.

J. D. PITTS, *Department of Biochemistry, University of Glasgow.*

Two mechanisms of cell–cell communication have evolved to allow the coordination of cellular functions in multicellular organisms. One mechanism depends on extracellular signal molecules (*e.g.* hormones, synaptic transmitters); the other depends on the formation of permeable intercellular junctions. Both mechanisms appear to be ubiquitous throughout the plant and animal kingdoms.

The permeability of junctions formed between animal cells is well established. Small ions and molecules are freely exchanged between the cytoplasms of all the cells in a coupled population, but macromolecules remain within the cells where they were synthesized (or their daughter cells which arise through division).[1,2] The exchange appears to take place by simple diffusion through specialized membrane structures (gap junctions) which bypass the normal permeability restrictions of the plasma membrane.[3,4]

In excitable tissues, permeable junctions can provide intercellular, low-resistance pathways for the propagation of electrical impulses. In non-excitable tissues, where the junctions are generally more numerous, they allow the equilibration of metabolite pools between cells. This can result in a tissue "phenotype" which is characteristic of a particular mixture of cells and which is different from the phenotypes of the separated cells.

In model systems in tissue culture, it has been shown that metabolic interactions through permeable junctions can result in the intercellular control of enzyme activities and cell proliferation.[5]

Gap junctions are found in most tissues, the only notable exceptions being skeletal muscle and some circulating cells. Tumour cells however, in all cases so far examined, have lost either the ability to form permeable junctions or the communication specificity of their normal counterparts.[6]

These difference between normal and tumour cells have led to the suggestion that defects in junctional communication might lead directly to the malignant state.[7] The evidence for and against this suggestion will be presented and discussed.

### REFERENCES

1 PITTS, J. D. & SIMMS, J. W. (1977) *Exp. Cell Res.*, **104**, 153.
2 LOEWENSTEIN, W. R. (1976) *Cold Spring Harbor Symp. Quant. Biol.*, **40**, 49.
3 GILULA, N. B., REEVES, O. R. & STEINBACH, A. (1972) *Nature*, **235**, 262.
4 CASPER, D. L. D., GOODENOUGH, D. A., MAKOWSKI, L. & PHILLIPS, W. C. (1977) *J. Cell Biol.*, **74**, 605.
5 PITTS, J. D. (1978) In *Intercellular Junctions and Synapses*, Eds J. Feldman, N. B. Gilula & J. D. Pitts. London: Chapman Hall. p. 63.
6 FENTIMAN, I. & TAYLOR-PAPADIMITRIOU, J. (1977) *Nature*, **269**, 156.
7 AZARNIA, R. & LOEWENSTEIN, W. R. (1977) *J. Membrane Biol.*, **34**, 1.

### THE SURFACE PROPERTIES OF INVASIVE AND METASTATIC TUMOUR-CELL POPULATIONS.

G. POSTE, *Department of Experimental Pathology, Roswell Park Memorial Institute, Buffalo, NY* 14263, *U.S.A.*

Evidence obtained in several laboratories over the last few years has shown that metastasis is not a random process in which each cell in a malignant primary tumour is capable of producing a metastatic lesion, but is instead caused by specialized subpopulations of cells within the primary tumour which are endowed with the properties needed to successfully complete all stages of the metastatic process.[1–3] Recognition that not all the cells in a malignant primary tumour possess the properties needed to metastasize, has major implications for the choice and design of experimental systems for the study of metastasis. Studies on unselected heterogeneous tumour-cell populations isolated from the primary lesion may offer little insight into the properties of metastatic cells, since the proportion of non-metastatic cells may be sufficiently high to prevent detection of properties which are unique to the metastatic subpopulation(s). Analysis of the malignant phenotype thus requires isolation and characterization of subpopulation(s) of malignant cells endowed with the full complement of properties needed for expression of metastatic behaviour.

The isolation of tumour-cell populations with invasive and metastatic properties has required the development of new methods whereby these cells can be reliably separated from cells lacking these behavioural traits. Recently, techniques have been devised which permit quantitative measurement of the ability of tumour cells to invade segments of chick chorioallantoic membrane, murine bladder wall and mammalian blood vessels cultured *in vitro*, and which also enable the invasive cell subpopulations to be reisolated.[4,5] Using these methods, a number of cloned lines of tumour cells with enhanced invasive and metastatic behaviour have been isolated,[5,6] and studies have begun to compare how these cells may differ from non-invasive and non-metastatic tumour cells present in the original parent cell population.

Recent studies on the adhesion of cells to collagen *in vitro*[7] and to the surface of blood vessels maintained *in vitro* in perfusion-culture, have revealed major differences in the behaviour of normal and tumour cells which may be relevant to the arrest of circulating tumour cells in the vascular bed and their subsequent extravasation to form micrometastases in the surrounding tissues. Normal cells adhere equally well to collagens of Types I, II, III or IV, but their adhesion is always serum dependent.[7] In contrast, metastatic tumour cells display a significantly higher affinity for adhesion to Type IV collagen and adhesion is serum independent.[7] Type IV collagen is found in the basement membrane of blood vessels, and these findings raise the intriguing possibility that the greater affinity of malignant cells for this substrate may aid their metastatic behaviour *in vivo* by facilitating cell arrest and extravasation. Other studies[8] on the adhesion of normal and tumour cells to blood vessels *in vitro* also support this view. Normal and malignant cells do not differ significantly in their ability to adhere to intact endothelium, but metastatic cells adhere more rapidly and in greater numbers to blood vessels denuded of endothelium. In the latter situation cell adhesion thus occurs directly to the basement membrane.[8] Perhaps of more importance is that the same differences in adhesion between normal and malignant cells have also been detected after minimal damage to the endothelium, in which the endothelial cells remain attached to the basement membrane but retract to create large intercellular spaces where the basement membrane is exposed. Since structural alterations of this kind can be induced *in vivo* merely by reduced blood flow, this raises the possibility that local alterations in blood flow created by the arrest of tumour cells in capillaries might predispose the vessel to adhesion of metastatic cells to the basement membrane.

Clinical[2,9] and experimental[3,10–17] observations showing a predilection of certain neoplasms for metastatic growth in specific organs suggest that the pattern of metastasis does not result from random arrest and growth of tumour cells, but reflects features of the circulating tumour cells, the host vasculature and/or the organ environment which cause selective growth in particular "target" organs. Convincing evidence that the properties of the tumour cells themselves can in part determine the pattern of metastasis has come from studies in which tumour-cell variants that localize preferentially in specific "target" organs have been isolated from the same tumour-cell line. Variants of the B16 mouse melanoma that localize preferentially to the lung,[3,19] the brain[3,17] or to the ovary have been isolated and found to display differences in their surface proteins as determined by lactoperoxidase-catalyzed iodination.[17] To study what role, if any, surface alterations play in determining the arrest and growth of these variants in particular target organs, Garth Nicolson and I have attempted to modify the arrest behaviour of these cells by manipulating the composition of their plasma membranes. This has been achieved by fusing plasma-membrane vesicles isolated from a B16 variant with high metastatic activity that localizes in the lung with a different B16 cell line which exhibits low metastatic activity and does not metastasize preferentially to the lung. Fusion of vesicles with the recipient cells results in a significant increase in the arrest of vesicle-modified cells within the lung, followed by increased formation of lung metastases.[19] These experiments indicate that: (1) plasma-membrane components present in the vesicles were able to modify the arrest behaviour of the recipient cells; and (2) surface properties are of importance in determining the arrest pattern of circulating tumour cells. Further studies to identify the plasma-membrane components involved in determining specific patterns of tumour-cell arrest are now in progress.

REFERENCES

[1] POSTE, G. (1977) In *Cancer Invasion and Metastasis: Biologic Mechanisms and Therapy*. Eds. S. B. Day *et al.* New York: Raven Press. p. 19.
[2] FIDLER, I. J., GERSTEN, D. M. & HART, I. R. (1978) *Adv. Cancer Res.* (in press).
[3] NICOLSON, G. L., BRUNSON, K. W. & FIDLER, I. J. (1978) *Cancer Res.* **38,** 4105.
[4] HART, I. & FIDLER, I. J. (1978) *Cancer Res.*, (in press).
[5] POSTE, G., FLOOD, M. & CHICHESTER, W. R. (1979) *Cancer Res.* (in press).
[6] HART, I. & FIDLER, I. J. (1979) *Cancer Res.* (in press).
[7] KLEINMAN, H. J., MURRAY, J. C., MCGOODWIN, E. B., MARTIN, G. R. & BINDERMAN, I. (1978) Supplement to *Calcified Tissue Abstracts*, p. 61.
[8] POSTE, G., FLOOD, M., DOLL, J. & BAIER, R. (1979) *Cancer Res.* (in press).
[9] WILLIS, R. A. (1972) *The Spread of Tumours in the Human Body*. 3rd Ed. London: Butterworths.
[10] PARKS, R. C. (1974) *J. Natl Cancer Inst.*, **52,** 971.
[11] PILGRIM, H. I. (1969) *Cancer Res.*, **29,** 1200.
[12] FIDLER, I. J. & NICOLSON, G. L. (1976) *J. Natl Cancer Inst.*, **57,** 791.
[13] GREENE, H. S. N. & HARVEY, E. K. (1964) *Cancer Res.*, **24,** 799.
[14] KINSEY, D. L. (1960) *Cancer*, **13,** 674.
[15] DUNN, T. B. (1954) *J. Natl Cancer Inst.*, **14,** 1281.
[16] SUGARBAKER, E. V., COHEN, A. M. & KETCHAM, A. S. (1971) *Ann. Surg.*, **174,** 161.
[17] NICOLSON, G. L. (1978) In *Biological Markers of Neoplasia: Basic and Applied Aspects*. Eds. P. Ruddon. Amsterdam: Elsevier. p. 227.
[18] FIDLER, I. J. (1973) *Nature*, **242,** 148.
[19] POSTE, G. & NICOLSON, G. L. (1979) *Proc. Natl Acad. Sci. U.S.A.* (in press).

### ABSTRACTS OF OPEN PAPERS

### CHARACTERIZATION AND PURIFICATION OF PLASMA MEMBRANES OF CULTURED HUMAN PANCREATIC ADENOCARCINOMA CELLS USING SPECIFIC ANTISERA AND IMMUNO-AFFINITY CHROMATOGRAPHY.

S. Pahlman, A. G. Grant, J. Herman-Taylor & I. Ljungsted-Pahlman, *Dept. of Surgery, St George's Hospital Medical School, London.*

An immunofluorescent study of the plasma-membrane antigens of an *in vitro* human pancreatic-cancer line was carried out using antisera to gastric and colonic cancer, normal omentum, $\beta_2$-microglobulin and gluteraldehyde-fixed whole cells. Persistent staining after suitable adsorption of the whole-cell antiserum suggested the presence of tumour-specific determinants. The expression of $\beta_2$-microglobulin on the cell surface was used to develop an immuno-affinity procedure for the further purification of plasma-membrane components by specific adsorption of the membranes to immobilized anti-$\beta_2$ antibody; desorption was achieved by detergent solubilization of retained membranes followed by PAGE characterization.

### SERUM β2-MICROGLOBULIN IN CANCER.

E. H. Cooper, R. A. D. Bunning, S. M. Illingworth, S. L. Haworth & S. A. Rashid, *Unit for Cancer Research, University of Leeds, Leeds.*

$\beta$2-Microglobulin ($\beta$2m) is an intrinsic part of HL-A antigens and also occurs in the free form on the surface of nucleated cells. In a large percentage of untreated non-Hodgkin's lymphoma (NHL) (68%) and Hodgkin's disease patients (55%) serum $\beta$2m was raised ($\geqslant$3·0 mg/l). The probability of raised $\beta$2m increased with more advanced disease, possibly indicating a relationship between $\beta$2m and tumour load, as does the observed lowering of $\beta$2m in some patients after chemotherapy. In remission, $\beta$2m levels tended to be normal, though a group of HNL patients was observed with persistantly high $\beta$2m, possibly due to residual disease. Most patients with chronic lymphocytic leukaemia had persistently raised $\beta$2m levels (76%) and there was no correlation between $\beta$2m levels and lymphocyte count. Elevated $\beta$2m was also present in 82% of patients with Burkitt's lymphoma. In bladder cancer patients with normal renal function as determined by serum creatinine levels, the frequency of raised serum $\beta$2m increased with tumour stage (15% T1, 57% T4) though a large number of patients had impaired renal function, making serum $\beta$2m measurement of little value. Few elevated $\beta$2m levels were found in prostatic cancer despite large tumour loads (30% raised in a no treatment M+ group). Whether this indicated low $\beta$2m production by tumour cells or little host reaction to this type of tumour is uncertain. $\beta$2m levels in choriocarcinoma were also seldom elevated, possibly a result of intensive chemotherapy and immunosuppression or a deficit of HL-A.

The support of physicians and surgeons in Yorkshire and elsewhere is acknowledged.

### CELL-SURFACE GLYCOPROTEINS AND GLYCOSAMINOGLYCANS (GA Gs) OF HUMAN SKIN FIBROBLASTS.

J. T. Gallagher, N. Gasiunas & S. L. Schor, *CRC Department of Medical Oncology, Christie Hospital & Holt Radium Institute, Manchester.*

Cell-surface glycoproteins play important roles in control of differentiation, cell interactions and probably the proliferative and metastatic abnormalities of tumour cells: regulation of synthesis and expression of specific glycoprotein ligands is clearly of importance in these processes. We have studied the effect of the cellular environment on membrane glycoproteins and GAGs of human skin fibroblasts by culturing cells on plastic or collagen-gel substrates. Confluent monolayers, incubated for 48 h with $^3$H-glucosamine and $Na_2{}^{35}SO_4$, were extracted with trypsin (0·5 mg/ml) when cultured on plastic, and by trypsin followed by collagenase (2 mg/ml) for cells on collagen gels which were not detached by trypsin. In cell-free extracts, heparin sulphate (HS) was the main sulphated GAG from cells on plastic with dermatan sulphate (DS) the minor component: on collagen gels, however, DS was more abundant than HS. Collagen-gel cultured cells were also enriched in cell-associated hyaluronic acid and a distinct acidic glycopeptide. We conclude that the cellular environment *in vitro* influences the synthesis and disposition of cell-surface glycoproteins and GAGs.

**PEANUT LECTIN RECEPTORS ON BREAST EPITHELIUM AND THEIR SIGNIFICANCE IN MAMMARY CARCINOMA.** R. A. NEWMAN, P. J. KLEIN & P. RUDLAND, *Imperial Cancer Research Fund, London, & Pathology Dept., University of Cologne, W. Germany.*

The Thomsen–Friedenreich (TF) antigen-specific lectin from peanuts was found to stain apical surfaces of normal human breast epithelium, when examined by fluorescence microscopy or autoradiography, but not myoepithelium or fibroblasts. Pre-treatment of histological sections with neuraminidase, however, increased staining over the total epithelial cell surface, showing that both free and sialic-acid-covered TF antigens are present, and is in contrast to recent reports that the TF antigen is a carcinoma-associated antigen. Receptors for the anti-A-like lectin from *Helix pomatia* gave a similar distribution. A number of benign and malignant breast tumours were studied, and showed distributions similar to normal tissue, although the intensity of staining increased with increasing tumour differentiation. Rat mammary gland from young, virgin, pregnant and lactating animals was also examined, as well as monolayer cultures of epithelial cells, and the amount of TF antigen found to correlate with the secretory status of the tissue. Peanut lectin can be used as a marker for breast epithelium but cannot be considered breast-carcinoma associated. Conventional immunotherapy using the TF antigen seems to offer poor expectations for success, although the effect of infiltrating tumours on cellular immunity remains to be investigated.

**ASPECTS OF APPLICATION OF SECONDARY-ION MASS SPECTROMETRY ON BIOLOGICAL MATERIAL: COMPARISON OF SPECTRA OBTAINED FROM GLIOMA AND GLIAL CELL SURFACES.** K. ZÄNKER,* D. STAVROU†, W. GERHARD‡, C. PLOG‡ & G. BLÜMEL*. **Institute for Exp. Surgery, TUM, †Institute for Neuropathology, Veterinary Medicine, LMU, Munich, ‡Dornier System GmbH.*

Changes in cell surfaces are believed to be involved in the regulation of cell growth. The idea that the surfaces of neoplastic cells differ from those of their normal counterparts has received support from various lines of research. To substantiate this view by a physical method, secondary-ion mass spectrometry (SIMS) was applied on N-methyl-N-nitrosourea-induced pleomorphic glioma and glial cell surfaces. SIMS allows researchers to monitor small molecules, of up to 10, probably more, atoms, which can be derived from various depths in the plasma membrane. All spectra of the positive ions showed a pattern of grouped peaks between 50 and 150 atomic mass units (u). A comparison of the relative distribution of the grouped peaks revealed that the decrease of the impulse heights towards higher u is steeper in the spectrum obtained from glia than from glioma. In addition, glial and glioma cells showed a characteristic positive ion peak at 148 u. The impulse height of the 148u peak was significantly lower within the glial cells, than the averaged impulse heights of the above grouped spectral lines. Evaluation of the 148 u in deeper layers of the membrane exhibited a small, continuous decrease of the impulse height within increasing membrane depths. The same experiment with glioma cells revealed a dramatic decrease of the 148 u in deeper membrane layers. These results suggest that (i) glioma cells express at the very topic of the cell surface a higher quantity of molecule(s) from which a 148 u can be fragmented than do glial cells and (ii) there is a different distribution of the original molecules. Further investigations should be undertaken to sort out whether SIMS might become a powerful tool for membrane-molecule fragmentation and, thus, to contribute to understanding neoplasia in terms of cell-surface transformation.

**FELINE ONCORNAVIRUS-ASSOCIATED CELL-MEMBRANE ANTIGEN (FOCMA).** J. C. NEIL, *Department of Veterinary Pathology, University of Glasgow Veterinary School, Bearsden Road, Bearsden, Glasgow.*

Although infection of cats with feline leukaemia virus (FeLV) is relatively common, leukaemia is a rare consequence. Recovery from FeLV infection is due to an immune response to viral antigens in which virus-neutralizing antibodies are produced. In addition, cats may make antibodies to a virus-induced non-virion antigen (FOCMA) which appear to protect them from the leukaemogenic effects of the virus. Antibodies to FOCMA were first recognised in cats with regressing feline sarcoma virus-induced tumours. Recently, FOCMA was defined as a tumour-specific cell-surface antigen which is induced in cats by FeLV infection and may be coded for by the FeSV genome. It is also found on leukaemic cells in cats with no evidence of FeLV infection (virus-negative leukaemias). We are attempting to isolate FOCMA from tumour-cell lines using immunoprecipitation and SDS polyacrylamide-gel electrophoresis. We thus hope to confirm the immunological evidence for this antigen and to assess its role in cell transformation and in the immunity of cats to FeLV and its leukaemogenic properties.

**CELL-CYCLE-RELATED CHANGES IN SURFACE MORPHOLOGY, VIRUS RELEASE AND VIRAL-ANTIGEN EXPRESSION.** S. Toth, *Department of Veterinary Pathology, University of Glasgow.*

Synchronized feline lymphoblastoid cells (FL 74) chronically infected with feline leukaemia virus (FeLV) have been examined, during the different phases of the growth cycle, by the combined means of cytoplasmic and viable-cell indirect membrane-immunofluorescence techniques (IFA), electron and immunoelectron microscopy (EM, IEM) and scanning electron microscopy (SEM).

Using IFA and EM techniques, it has been found that virus production and viral protein expression are cell-cycle dependent and occur near mitosis.

In order to establish a more precise timing of the above events, immunoferritin labelling and SEM of synchronized cells were performed.

Several distinct changes were found in surface morphology, which correlated with the subdivisions of the cell-cycle, and analysis of the surface morphology, ultrastructure, the number of budding C-type particles, the distribution and intensity of ferritin-labelled antigenic sites allowed the identification of individual cells in the cell cycle.

**CELL-SURFACE ANTIGEN AND RECEPTOR EXPRESSION ON THYMIC LYMPHOMAS OF THE GR MOUSE STRAIN AND ON SOMATIC CELL HYBRIDS WITH CHINESE HAMSTER (E36) CELLS.** J. Hilgers, J. Hilkens & A. Colombatti, *Division of Genetics, The Netherlands Cancer Institute, Amsterdam.*

Transplanted ascitic thymic lymphomas of the GR mouse strain (GRSL) were compared with thymocytes, for cell-surface antigens and receptors. Three types of GRSL's could be distinguished on the basis of Ly-antigen expression. The majority of leukaemias were negative for Ly1.2 and Ly2.1, some were positive for Ly1.2 only and so far one was Ly2.1 positive. All 3 types of leukaemia express antigens not present on thymocytes, such as the Mammary Tumour Virus-induced MLr antigen (residing on the MTVgp52 envelope protein) and "extra" TL antigens. These leukaemias express Thy1.2 antigen in a low and variable way, due to "masking" by carbohydrates. This is also the case for the cholera-toxin receptor (presumably the GM1 ganglioside). Some antigens and receptors are expressed better on GRSL cells than on thymocytes, *e.g.* H-2K and H-2D antigens and the Rauscher Leukaemia virus gp70 receptor.

In order to study expression of these cell-surface markers as a function of the "differentiated" state, somatic cell hybrids segregating mouse chromosomes were generated between GRSL and Chinese hamster E36 lung fibroblasts. Expression of H-2 antigens and the 2 receptors, not present in E36, segregated in the hybrids. Some, but not all, fibroblast-like hybrids retaining chromosome 17 with the TLa locus, still expressed the "extra" TL surface antigens, but so far all clones retaining chromosome 9 with the gene for Thy1.2 did not express this cell-surface antigen. Expression of the MTV, both at the cell surface and in the cytoplasm (radioimmunoassays for MTVp27gag and MTV-gp52env), was absent in all clones, even if the full complement of MTV-DNA copies of the GRSL parent were still present.

**CIRCULATING TUMOUR-SPECIFIC FACTORS: DIAGNOSIS OF PROGRESSIVE GROWTH OF A TRANSPLANTED RAT HEPATOMA.** J. G. Bowen, D.

HANNANT & R. W. BALDWIN, *Cancer Research Campaign Laboratories, The University, Nottingham.*

The inhibition of membrane-immunofluorescence assay has been used to quantitate tumour-specific antigens (TSA) in the circulation of rats bearing the chemically induced transplanted hepatoma D23. Rats were inoculated either s.c. or i.m. with defined numbers of viable hepatoma D23 cells. Free circulating TSA was detected at about Day 7–10 in rats challenged with $5 \times 10^5$ D23 cells s.c. or $4 \times 10^4$ cells i.m., and the appearance of free TSA coincided with the development of palpable tumour. This initial peak of free antigen was followed by a rise in the levels of immune-complexed antigen during the later stages of tumour growth after the induction of a tumour-immune antibody response. When the challenge dose of hepatoma D23 cells was close to the minimum inoculation required for progressive tumour growth ($5 \times 10^2$ cells s.c.; $10^2$ cells i.m.), there was a delay in the appearance of palpable tumour (about 20 days after inoculation), ~11 days beyond the time when free TSA was detected. Inoculation of $\gamma$-irradiated (15,000 R) non-viable hepatoma D23 cells failed to produce evidence of free-circulating TSA when it was detectable in rats inoculated with viable tumour cells. These results indicate that the presence of free hepatoma D23 antigen in the serum of rats is diagnostic for tumour growths, when the challenge inoculum is near to the minimum inoculum. Furthermore, the presence of antigen is a function of the presence of viable tumour cells in the host.

**A NEW HUMAN EPITHELIAL-MEMBRANE ANTIGEN (EMA).** E. HEYDERMAN*, K. STEELE† & M. G. ORMEROD†, **Ludwig Institute for Cancer Research, Royal Marsden Hospital, and* †*Institute of Cancer Research, Royal Cancer Hospital, Sutton, Surrey.*

We have demonstrated a new epithelial-membrane antigen (EMA) using antisera raised against defatted human cream and an indirect immunoperoxidase technique on routinely processed and fixed tissue sections and on monolayers. The antigen is localized on a wide variety of epithelia, including normal and neoplastic mammary tissue, adenocarcinomas from various sites, several types of normal secretory epithelium and the distal tubules and collecting ducts of the kidney.

Although the distribution of the antigen is widespread, it is highly selective, apparently localized to membranes associated with a secretory function and adenocarcinomas. We have shown that EMA is different from carcinoembryonic antigen (CEA), normal cross-reacting antigen (NCA), $\beta$-oncofoetal antigen (BOFA), pregnancy-specific glycoprotein ($\beta_1SP_1$) and the blood-group substances A, B, Lewis a and Lewis b, by comparing their distribution in various tumours and some absorption studies. Like CEA in poorly differentiated tumours, the antigen is frequently cytoplasmic in distribution.

Since the antigen is carried by a variety of adenocarcinomas its demonstration in minute foci of malignant cells in the marrow and lymph nodes is but one example of a possible role in the diagnosis and differential diagnosis of malignant disease.

**LYMPHOCYTE-STIMULATION BY $Ia^+$ AND $Ia^-$ ACUTE MYELOID LEUKAEMIAS AND CELL LINES.** G. M. TAYLOR, W. FERGUSSON & R. HARRIS, *Dept. of Medical Genetics, St Mary's Hospital, University of Manchester.*

Lymphocyte stimulation by $Ia^+$ and $Ia^-$ leukaemias and cell lines was studied.

Certain of the leukaemias and cell lines stimulated allogenic lymphocytes *in vitro*; others did not. Some of the non-stimulating leukaemias were found to express Ia alloantigen by using poly-specific human anti-Ia serum, and the p28,33 antigen detected by rabbit antiserum. Variations were found in different leukaemias in the proportion of $Ia^+$ cells, and the strength of antigen on individual cells.

Lack of stimulation by $Ia^+$ leukaemias could not be attributed to poor viability, or dependence upon 2-mercaptoethanol. The relationship between lymphocyte-stimulating and Ia antigens was assessed by *in vitro* blocking of lymphocyte stimulation with anti-Ia serum. Such parallel studies of lymphocyte-stimulating and Ia antigens should show how lymphocytes respond to cell interaction (Ia) antigens on leukaemic-cells.

**SINGLE-CELL ANALYSIS OF CELLS CARRYING LEUKAEMIA-ASSOCIATED ANTIGENS IN NORMAL AND REGENERATING MARROW.** G. JANOSSY, M. F. GREAVES & F. J. BOLLUM, *Dept. Immunology, Royal Free Hospital and ICRF, London.*

The leukaemic blast cells in the common form of acute lymphoblastic leukaemia and in most cases of so-called "lymphoid" blastic transformation of $Ph^{+}$ chronic myeloid leukaemia show a characteristic phenotype. These cells react with anti-ALL serum, carry Ia-like antigens and contain, in the nucleus, the enzyme terminal transferase. Antibodies to these antigenic structures have been developed and used in combination in order to show that a few small cells with lymphoid appearance can be found in regenerating marrow. These marrow samples were taken from patients who had had cytotoxic therapy for malignancies other than lymphoid leukaemia or from non-leukaemic patients. These cells could be the early haemopoietic stem cells from which the malignancy of the same phenotype arises. The clinical application of this single-cell analysis will be discussed.

**MEMBRANE SPECIALIZATION IN CELL-SUBSTRATUM ATTACHMENT SITES.** T. D. ALLEN, M. J. BRITCH & C. J. HARRISON, *Department of Ultrastructure, Paterson Laboratories, Christie Hospital & Holt Radium Institute, Manchester.*

The attachment of cells to substrata has been characterized to date using the interference reflection microscope, with correlation in the high-voltage electron microscope (Heath & Dunn, 1978, *J. Cell Sci.*, **29**, 197). These studies have indicated areas of attachment between the cell underside and the substratum that are limited to localized "focal contacts", with some evidence for the insertion of actin-filament bundles in these regions (Abercrombie *et al.*, 1971, *Exp. Cell Res.*, **67**, 359). Studies in these laboratories using an epithelial cell line from liver observed during the process of spreading and detachment, have characterized peripheral regions of the cytoplasm which have been interpreted as specialized cell–substratum attachment sites. These areas have been investigated by whole-mount TEM and SEM of the same cell, and also underside replicas produced by freeze fracture. Ruthenium-red staining of the whole-mount preparations has also illustrated the production and involvement of cell-coat material in these regions. The regions of attachment are characterized by a reticulate appearance of the peripheral cytoplasm, in roughly circular regions of 5–10 $\mu$m in diameter. These areas appear in the lamellar regions of spreading epithelial cells, or leading edges of fibroblasts, and appear concomitant with a loss of membrane ruffling. Initially these regions show no insertion of microfilaments, but an association may be developed subsequently. As well as becoming the initial sites of membrane alteration during settling and locomotion, these areas are also the most resistant to the effects of trypsin and EGTA and may consequently correlate with the substratum-attached material (SAM) which remains after cell detachment.

**FACTORS INFLUENCING CELL ATTACHMENT TO NATIVE COLLAGEN FIBRES AND DENATURED COLLAGEN FILMS.** S. L. SCHOR, J. COURT & J. PRUDHOE, *CRC Department of Medical Oncology, Manchester University & Christie Hospital & Holt Radium Institute, Manchester.*

The majority of previous reports dealing with cell attachment to collagen have used collagen films extracted with urea as a substratum; these studies have demonstrated that cell attachment to such films is dependent on a high-mol.-wt glycoprotein (variously known as LETS, fibronectin, CSP, CAP etc.) found both in serum and on the surface of normal fibroblasts. In the present communication, we wish to report that only cell attachment to *denatured* collagen films is dependent on this glycoprotein, and that cell attachment to native collagen fibres is mediated by a different (serum-independent) mechanism.

This conclusion is supported by observations comparing the kinetics and extent of cell attachment: (a) to various types of collagenous substrata; (b) following treatment of the cells with either EGTA only or EGTA+trypsin (to remove the cell surface LETS) and (c) in the presence and absence of foetal calf serum.

**QUANTITATIVE MORPHOLOGICAL CHANGES AT THE EPITHELIAL-CONNECTIVE TISSUE JUNCTION DURING ORAL CARCINOGENESIS.** F. H. WHITE & K. GOHARI, *Department of Oral Pathology, University of Sheffield.*

The basal-lamina complex together with the hemidesmosomes are thought to be responsible for epithelial–connective-tissue adherence. Ulstrastructual studies have demonstrated marked alterations in this region in both human neoplastic development and in chemical carcinogenesis. This study was designed to investigate some of these alterations using quantitative methods. Biopsies were obtained from 7,12 dimethylbenz(α)anthracene (DMBA)-treated pouches which demonstrated defined histological features corresponding to hyperplasia (H), dysplasia (D) or carcinoma (C). Untreated pouch epithelium served as control (N). Stereological intersection counting procedures were used to estimate relative surface parameters for hemidesmosome and basal plasma membrane (HD/BM) hemidesmosome and lamina densa (HD/LD) lamina densa and basal membrane (LD/BM) and mean hemidesmosomal diameter ($\bar{\Delta}$HD). Results were as follows:

HD/BM: N=0·40; H=0·34; D=0·28; C=0·13.

HD/LD: N=0·40; H=0·39; D=0·37; C=0·30.

LD/BM: N=0·98; H=0·88; D=0·76; C=0·42.

$\bar{\Delta}$HD: N=0·23; H=0·23; D=0·22; C=0·20.

The results indicate a progressive decrease of both hemidesmosomes and basal lamina during carinogenesis. Quantitative evaluation of these structures in human neoplastic development may prove to be useful diagnostic and prognostic indicators.

**THE COMMUNICATION PATTERN OF CULTURED HUMAN BREAST CELLS.** I. FENTIMAN & J. TAYLOR-PAPADIMITRIOU, *ICRF, London.*

The pattern of direct communication of cells derived from the human breast has been examined in culture, using the $^3$H-nucleotide transfer method. Normal human mammary epithelium from a variety of sources has been found to demonstrate selectivity in communication. Thus although HumE communicate with homologous cells, no transfer of nucleotide occurs from HumE donors to fibroblast recipients, including those derived from the human breast (Fentiman *et al.* (1976), *Nature*, **264** 760).

This work was extended to human breast-cancer cells, either primary cultures obtained directly from tumours, or established lines derived from either primary carcinomas or pleural-effusion metastases. Primary tumours yielded 2 epithelial populations, one of which was indistinguishable morphologically from normal mammary epithelium. The other cells, designated HumE$^1$, arose from about one third of primary and metastatic carcinoma, and may represent the actual malignant cells.

HumE$^1$ as donors showed 2 patterns of communication, being either non-communicators, or non-selective communicators, *i.e.* capable of transferring nucleotide to any cell type capable of communication. A similar pattern was shown by established lines (Fentiman & Taylor-Papadimitriou (1977), *Nature*, **269**, 156). This loss of selectivity may confer survival benefit on metastizing cancer cells. Further, this change in pattern of communication may serve as a parameter for distinguishing normal from malignant human breast epithelium in culture.

**NEURAMINIDASE-MEDIATED MODIFICATION OF METASTATIC DISEASE BY BLOOD LYMPHOCYTES CULTURED WITH PRETREATED TUMOUR CELLS.** E. WATKINS, JR., L. L. ANDERSON, O. L. BARALT, R. B. MAHONEY, S. C. HOLLIS & G. J. HEATLEY, *Sias Research Laboratory, Lahey Clinic Foundation, Boston, Mass., USA.*

Diethylstilboesterol - induced oestrogen - sensitive mammary carcinoma MT/W 449 (syngeneic in WF rats) shows 100% metastasis to lung and lymph nodes after amputation of hind limbs bearing trocar tumours $\geq$12 mg in male WF/Sch rats (71/71 animals, MST after implantation 114 days, range 80–197). I.p. injection 6 days after amputation of 1–40 $\times 10^6$ Methocel-Hypaque peripheral-blood lymphocytes (PBL) cultured 6 days with MT/W449 tumour cells pretreated with *V. cholerae* neuraminidase (VCN) significantly prolonged actuarial cohort survival (MST 129

days, 11/62 animals (17·7%) disease-free beyond longest control survival, range 220–534 days, $P=0{\cdot}0006$). Optimal prolongation of survival was seen after injections of $20\text{–}30\times10^6$ PBL cells. No such protective effects were seen after similar injection of PBL cultured alone. PBL cultured with sham-pretreated MT/W 449 cells, or sham-pretreated or VCN-pretreated MT/W 449 cells injected immediately after amputation or after 6 days culture.

The immunogenic effect of VCN-pretreatment of tumour cells may be related to tumour-cell-bound enzymatically active VCN co-ligating with PBL cell-surface glycoprotein, as indicated by increased tumour-cell–PBL rosette formation and biphasic release of additional free sialic acid when pretreated washed tumour cells are mixed with PBL. Non-specific inhibition of immunogenic effect by acute-phase serum sialoglycoprotein may be related to competition of such proteins and PBL surface glycoproteins for tumour-bound VCN enzymatic binding sites.

(USPHS-NCI Grant CA 18938)

**STUDIES ON METASTATIC SPREAD OF PRIMARY TUMOURS.** D. Tarin & J. E. Price, *Department of Histopathology, Royal Postgraduate Medical School, Hammersmith Hospital, London.*

A model has been developed for studying the capability of cells from primary mammary tumours to establish colonies in distant organs. The aim was to avoid the use of repeatedly transplanted tumours or serially propagated tumour cell lines. The model involves the intravascular inoculation of disaggregated tumour cells into autochthonous and syngeneic recipients. The results show that the colonization potential of cells from a given tumour is consistent between the animals of an inoculated batch. Also, the findings are similar in the autochthonous host and the syngeneic recipients. Tumours vary in their metastatic colonization potential and can be classified into high and low groups. These findings indicate that:

(i) A significant proportion of mammary tumours (40%) are capable of establishing colonies in distant organs even though the incidence of metastatic spread of these tumours in the undisturbed animal is almost zero.

(ii) The colonisation potential of the tumours is an intrinsic property of the tumour cells rather than of the host.

The model will now be used to study cellular properties which favour colonization of distant organs.

**A COMPARISON OF THE SURFACES OF TUMOUR CELLS ISOLATED FROM A METASTASIZING AND A NON-METASTASIZING LYMPHOSARCOMA.** G. A. Turner, A. L. Latner, D. Guy & G. V. Sherbet, *Department of Clinical Biochemistry, Royal Victoria Infirmary, Newcastle upon Tyne.*

Collagenase treatment isolates single cells from solid tumours without substantial disruption of the cell surface (Guy *et al.*, 1977, *Br. J. Cancer*, **36,** 166). Using this preparative procedure, surface properties of cells from a metastasizing (ML) and a non-metastasizing (NML) lymphosarcoma (Carter, 1966, *Am. J. Path.*, **49,** 637) were compared in respect of adhesion to polystyrene-coupled lectins (Edelman *et al.*, 1971, *Proc. Natl Acad. Sci.*, **68,** 2153); cytopherometry (Latner & Turner, 1974, *J. Cell Sci.*, **14,** 203); isoelectric focusing (Sherbet & Lakshmi, 1973, *Biochim. Biophys. Acta*, **298,** 50); and radioiodination (Guy *et al.*, 1977). Primary ML and NML cells gave identical surface radiolabelling and cell-adhesion patterns, isoelectric points of 4·65 and 4·49 pH units respectively, and electrophoretic mobilities of 2·02 and 1·46 $\mu$m $sec^{-1}$ $V^{-1}$ cm respectively. No differences could be detected in surfaces of primary and secondary cells from the ML tumour. These data suggest that the acquisition of metastatic capability is accompanied by a general rather than a specific change in the cell-surface profile.

### ABSTRACTS OF POSTERS

### REPLICAS OF THE CELL SURFACE AND UNDERSIDE MEMBRANE.

M. J. Britch & T. D. Allen, *Department of Ultrastructure, Paterson Laboratories, Christie Hospital and Holt Radium Institute, Manchester.*

Recently several methods for *in situ* freeze fracturing of cultured cell monolayers have been reported. The method of Pauli *et al.* (1977, *J. Cell Biol.*, **72,** 763) has been modified to produce replicas of the cell surface and underside.

Cells were cultured to confluency on plastic coverslips. Two coverslips drained of excess medium, were sandwiched in a drop of polyvinyl alcohol. The cellular sandwich was then quenched in melting $N_2$ at 63K. The two coverslips were fractured apart under liquid $N_2$ and transferred to a modified cold stage in the NGN FE600 freeze-etch machine. The cells were etched for 5 min in a working chamber evacuated to less than $1{\cdot}33 \times 10^{-4}$ $N/m^2$.

Replicas of the cell surface and the cell underside were collected. Underside replicas produced by shearing of the frozen cells from the substrate demonstrate the relationship of microfilament bundles to the underside membrane. Areas of membrane perturbation, widely distributed in the underside membrane, have been interpreted as sites of membrane–substrate interaction, disturbed by shearing of the cells from the substrate. They correspond closely to the adhesion sites observed in whole-cell TEM.

Replicas of the cell surface of unfixed frozen cells were also examined. The surface morphology of rounded-up mitotic fibroblasts was basically similar to that of chemically fixed, coated cells observed in the SEM. High-resolution TEM has revealed considerable surface detail. The surface is highly folded and is frequently thrown into ridges. Microvilli, clearly seen as modified surface folds, are often associated with these ridges.

### THE METHOD OF CELL ROUNDING IN THE PRESENCE OF TRYPSIN IS CELL-SHAPE DEPENDENT.

C. J. Harrison & T. D. Allen, *Department of Ultrastructure, Paterson Laboratories, Christie Hospital and Holt Radium Institute, Manchester.*

The exposure of cells to trypsin revealed the series of events leading to the assumption of a spherical morphology prior to cell detachment from the substratum. It was seen that the method of cell rounding was cell-shape dependent.

Fibroblasts possess points of adhesion at the tips of their polar regions. Continued trypsin-induced cytoplasmic contraction applied stress to the adhesion points until attachment was released at each pole in turn. This produced recoil of the polar regions under elastic tension to produce spherical cells with a blebbed surface morphology.

Cells of epithelial outline assumed a spherical shape with a microvillous cell surface by slow retraction of the cytoplasm around the entire cell periphery. An epithelial outline was maintained throughout the rounding-up process. The alteration of cell shape as fibroblasts progressed through the cell cycle by transformation or induced by various experimental methods showed the same results.

### VISUAL MARKERS FOR CELL-SURFACE RECEPTORS IN THE SEM.

S. L. Goodman, G. M. Hodges & D. C. Livingstone, *Imperial Cancer Research Fund, London.*

The visual detection and localization of specific molecules on the cell surface by SEM is possible using probes such as colloidal gold, methacrylate latex spheres and defined silica spheres. This approach is being applied to studies designed to explore and correlate the expression and topographical distribution of cell-surface components with cell-surface features in normal and neoplastic tissues. Work is currently in progress to evaluate the characteristics, preparation and further applicability of these probes and their ligand–marker conjugates.

### CELL-SURFACE MORPHOLOGY ASSOCIATED WITH CYTODIFFERENTIATION AND NEOPLASTIC TRANSFORMATION OF UROTHELIAL TISSUES.

G. M. Hodges, *Imperial Cancer Research Fund, London.*

There is clear evidence that urothelia maturation is associated with a series of welldefined changes in the architectural organization of the urothelial cell surface. This is revealed as progressive development and fusion of small globular microvilli into the characteristic convoluted angular membrane infolds of the fully differentiated superficial urothelial cell. By contrast, this surface-pattern sequence is modified in chemical-carcinogen-treated urothelium or in human bladder tumours, frequently in association with the development of microvilli of a pleomorphic nature. Current evidence suggests that such changes in surface properties should be of value as an indicator of neoplastic transformation.

**PLASMA MEMBRANES AND CELL CYCLE IN MOUSE PAROTID GLANDS.** R. O. LOPEZ-SOLIS & J. P. DURHAM, *Department of Clinical Oncology, University of Glasgow.*

Isoproterenol (IPR), a synthetic catecholamine, induces cell proliferation in acinar cells of mouse parotid glands when injected i.p. The role of the plasma membrane in the induction of DNA synthesis is being studied. Plasma membranes from this tissue were isolated by differential and discontinuous sucrose-gradient centrifugation at different stages of the cell cycle, and the activity of different enzymes known to be "markers" for these plasma membranes were studied in order to have an additional element of structural and functional analysis of this compartment of the cell cycle. The results show that between 0 and 28 h (injection of IPR and onset of DNA synthesis respectively) the specific activities of these plasma-membrane markers change continuously, which could reflect an active role of the cell surface during the cell cycle. Moreover, the description of changes in specific molecules with better known physiological functions could help to understand the participation of the cell surface between the stimulation and onset of DNA synthesis.

**PROTEINS AND GLYCOPROTEINS ON HUMAN MELANOMA CELLS.** G. P. ROBERTS & D. L. JONES, *University Department of Surgery, Welsh National School of Medicine, Cardiff.*

Characterization of the cell-surface components of tumour cells is important for an understanding of the role these materials may have in malignancy. We have used iodination with $^{125}I$ in the presence of lactoperoxidase to label the cell-surface proteins and glycoproteins of melanoma cells. The labelled components were separated by SDS electrophoresis on 5–22·5% polyacrylamide gradient gels and detected by autoradiography. Fractionation of extracts of the labelled cells by affinity chromatography on immobilized lectins indicated that both proteins and glycoproteins were labelled. Identification of some of the labelled bands was achieved by addition of immune sera to extracts of the iodinated cells followed by examination of the immune complexes retained on *Staphylococcus aureus*. In this way proteins originating from foetal calf serum used in the culture medium and glycoproteins cross-reacting immunologically with those of erythrocytes, lymphocytes and fibroblasts were detected on the melanoma cell surface.

**ISOLATION AND CHARACTERIZATION OF A MEMBRANE ANTIGEN FROM ACUTE LYMPHOBLASTIC LEUKAEMIA.** R. SUTHERLAND, J. SMART & M. F. GREAVES, *Imperial Cancer Research Fund, London.*

We have previously documented the presence of a cell-surface antigen found in acute lymphoblastic leukaemia, chronic myeloid leukaemia in blast crisis and some lymphomas (Greaves, *et al.* (1975), *Clin. Immunol. Immunopath.*, **4,** 67; Roberts *et al.* (1978) *Leukaemia Res.*, **2,** 105). This antigen is also expressed on established cell lines derived from these leukaemias (Minowada *et al.* (1978) *Natl Cancer Inst.*, **60,** 1269) which we have used as a convenient cell source from which to isolate the antigen (Sutherland *et al.* (1978) *Leukaemia Res.*, **2,** 115). Cells were labelled with either $^{125}I$ by the lactoperoxidase technique or with $^{35}S$ methionine or $^{3}H$-leucine for metabolic labelling. Total cell extracts, purified cell membranes or culture supernatant were used as a source of antigen, membranes being solubilized in NP-40 detergent. Sepharose-lentil and Sepharose-ricin lectin columns were used to purify glycoproteins and the ALL antigen was affinity purified from these by precipitation with anti-ALL antibodies on *S. aureus*. Antigen eluted from the bacteria was run in polyacrylamide-gel electrophoresis and its position and apparent mol. wt determined after autoradiography or fluorography. The results show that the ALL antigenic determinant is released from cells on a single glycosylated polypeptide with an apparent mol. wt of 100,000. Membrane-associated ALL antigen may be disulphide linked to a smaller component, giving an apparent mol. wt on non-reducing conditions of 130,000. The molecule is homogeneous by isoelectric focusing (5·8).

**BIOPHYSICAL CHANGES OF CELL MEMBRANE DURING THE GROWTH OF AN ASCITIC TUMOUR.** P. Bischoff, F. Robert & M. Donner, *INSERM, Unit of Experimental Cancerology and Radiobiology, Plateau de Brabois, 54500 Vandoeuvre-lès-Nancy, France.*

There is a considerable interest in studying the electrical charge of cell surfaces, since it is accepted that the interactions of cancer cells with host immunocompetent cells are in part determined by the physicochemical nature of the cell periphery (Sanford & Codington (1971) *Tissue Antigens*, **1**, 153). An ascitic tumour (SEWA) induced by polyoma virus in A.SW mice was analysed *in vivo* as well as *in vitro* with regard to the electrophoretic mobility (EPM) which may be considered as a reliable criterion of surface charge. After the i.p. inoculation of $10^5$ cells, the EPM decreased up to 14th day (1·30 μm/sec/Vcm). Then the mobility gradually increased with the age of the tumour. The EPM was 1·58 μm/sec/Vcm for 28-day-old tumours. Chemical modification of cell surface with maleic anhydride and neuraminidase showed important variations of ionized chemical groups in 14- and 28-day-old tumours. Amino groups showed a drastic decrease during the late phase of tumour growth. In contrast, the number of α-carboxyl groups of N-acetyl neuraminic acid markedly increased. On the other hand, incubation with antisera of mice immunized against SEWA are without effect on electrophoretic mobilities. When SEWA was maintained *in vitro*, the EPM reached a peak 24 h after renewal of the medium. This increase was not seen when cells were treated with cytosine arabinoside. Though EPM variation in this case may be interpreted on the basis of cell-proliferation kinetics, the interpretation of *in vivo* results is more complex. These results led us to suggest that changes observed *in vivo* might be the consequence of different independent parameters. Besides intrinsic factors such as cell kinetics, extrinsic factors are able to modify pericellular environment. We have considered the possibility of cell coating by immunoglobulins present in ascitic fluid.

**COMPUTER ANALYSIS OF IMMUNOFLUORESCENCE DATA OBTAINED WITH FLOW CYTOMETRIC SYSTEMS.** J. V. Watson, T. Pearson & A. Ziegler, *M.R.C. Laboratories of Clinical Oncology and Molecular Biology, The Medical School, Cambridge.*

A computer model has been developed to analyse data obtained from cell-surface-marker labelling using flow systems. Peripheral lymphnode cells of C57BL/10 and BALB/c mice were treated with serial dilutions of a fluorescinated monoclonal anti-mouse "IgD" reagent and analysed in 2 different flow systems, a model 4800A Cytofluorograf (CFG) and a Fluorescence Activated Cell Sorter (FACS). Comparisons of the predicted proportions of labelled cells from the computer analysis with those from conventional integration above and below an arbitrary level set "by eye" in each instrument, permit the following conclusions. (1) Due to the high coefficient of variation and low laser power of this model of the CFG, an accurate quantitation of data can only be obtained by computer analysis. (2) Both the conventional integration above and below an arbitrary response level set "by eye" and the computer analysis gave similar results with the FACS, but the data indicate that the discriminating level should be checked by computer if the conventional method is chosen. (3) Whichever instrument, or method of data evaluation is chosen, it is essential to carry out experiments with serial dilutions of the fluorescent antiserum to determine the optimum experimental conditions. (4) It is necessary to determine the fraction of non-specifically labelled cells in the sample, even with monoclonal reagents.

**CELL SURFACE CHANGES IN CANINE MAMMARY CARCINOMA.** R. W. ELSE, *Department of Veterinary Pathology, University of Edinburgh,* D. HANNANT, *Cancer Research Campaign Laboratories, University of Nottingham.*

Existing knowledge of spontaneous canine mammary carcinomas indicates that although 40–50% of tumours can be surgically ablated, other histologically similar neoplasms are more aggressive, with a greater propensity to metastasis. There is also a proportion (9%) of benign tumours which undergo malignant transformation. The reasons for the apparent differences and changes in relative immunogenicity of these tumours is unknown. Recent studies (Hannant & Else (1978) *Vet. Rec.*, **103,** in press) have shown that dogs with solid-type mammary carcinomas have circulating immune complexes with a tumour-associated antigen component. In addition, TEM and SEM of primary tumours, metastases, cultured cells and tumour-cell suspensions of solid-type carcinomas demonstrated cytoplasmic and surface-membrane changes. The latter are characterized chiefly by extensive microvillous proliferation and absence of glycocalyx compared with normal epithelial cells. These surface changes may be of significance in relation to the metastatic potential of the tumours. They may also be a morphological expression of different antigenicity and therefore reflect relative tumour immunogenicity.

**ANTIGENS OF 3-METHYLCHOLANTHRENE-INDUCED RAT SARCOMAS: HETEROGENEITY WITHIN PRIMARY TUMOURS AND DIFFERENCES BETWEEN PRIMARIES AND METASTASES.** M. V. PIMM, *Cancer Research Campaign Laboratories, University of Nottingham.*

It was previously found that tumour recurrences at the site of excision of primary MCA-induced rat sarcomas were antigenically distinct from the resected primary tumour (Pimm & Baldwim (1977) *Int. J. Cancer*, **20,** 37). This may reflect development of dormant neoplastic cells after surgical removal of an antigenically homogeneous primary tumour, or alternatively true antigenic heterogeneity within the primary growth, as suggested by the earlier work of Prehn (1970, *J. Natl Cancer Inst.*, **45,** 1039).

To examine these possibilities further, in the present study the antigenicity of *in vivo* lines established from opposite poles of primary sarcomas have been compared, and with one primary tumour-bearing animal the antigenicity of lines established from peritoneal, renal and pulmonary metastases have also been examined. Sublines from 4 primary tumours cross-reacted, so that immunization against one line conferred protection against the other. With a 5th tumour, neither subline was antigenic, although they had similar growth characteristics. In contrast, sublines from 3 further sarcomas were antigenically distinct, so that immunization against the subline from one pole failed to protect against the other. Furthermore, with one primary sarcoma, while the line from a peritoneal metastasis cross-reacted with lines from the primary tumour, neither of these cross-reacted with lines from pulmonary and renal metastases, although these latter were cross-reactive with each other.

These studies demonstrate that antigenic heterogeneity can exist within established primary tumours, and that metastases may be antigenically distinct from the primary tumour.

**SURFACE ANTIGENS ON NORMAL AND TRANSFORMED LYMPHOID CELLS.** C. M. STEEL, V. VAN HEYNINGEN, D. L. DEANE & B. B. COHEN. *MRC Clinical and Population Cytogenetics Unit, Edinburgh,* & C. H. W. HORNE & A. W. THOMSON, *Department of Pathology, Aberdeen University Medical School.*

Most long-term established B lymphoid cell lines are potent activators of allogeneic or autochthonous T lymphocytes in mixed lymphocyte culture (MLC). The property is not dependent on the presence of EB virus in the transformed lines. Untransformed B cells also stimulate in MLC, though much more weakly.

One "variant" EBV-carrying B cell line ($EB_1$) is markedly deficient in MLC stimulating capacity although it expresses surface HLA (including DR) antigens. A rabbit antiserum raised against human B lymphoblastoid cell (DAUDI) membranes and then serially absorbed with T cell lines and $EB_1$ cells, displays only weak residual complement-dependent cytotoxicity (*i.e.* it has little conventional anti-DR activity). Nevertheless, IgG from this antiserum binds to the surface of those B-cell lines capable of stimulating in MLC, as can be demonstrated in immunofluoresence and EM immuno-ferritin labelling tests. The antibody can also be shown to inhibit MLC reactions, but does not impair lymphocyte responses to soluble mitogens. The antigen(s) which it detects appear to be expressed on untransformed B lymphocytes as well as transformed B lymphoblastoid cells, though there may be important quantitative differences.

Competitive blocking tests indicate a very close association between the antigens detected by the above antiserum and pregnancy-associated alpha$_2$ glycoprotein ($\alpha_2$-PAG) which has been implicated in the regulation of lymphocyte-mediated immune reactions.

**THE ASSOCIATION OF HOST IMMUNOGLOBULINS WITH SOLID TUMOURS *IN VIVO*.** K. James, J. Merriman & Y. Bessos, *Department of Surgery, University of Edinburgh Medical School.*

In order to ascertain whether circulating antitumour antibodies are capable of interacting with tumour cell-surface antigens *in vivo* we have developed techniques to detect Ig on freshly excised tumours. The techniques employed include a direct and indirect radioimmune antiglobulin technique which permits a semi-quantitative assessment of tumour-associated mouse Ig, and a competitive radioimmunoassay technique which enables the precise quantitation of mouse Ig classes and sub-classes in NP 40 extracts of freshly excised tumours. Using these procedures we have found that the amount of Ig associated with tumours *in vivo* is dependent upon the immunogenicity of the tumours, the source of the initial tumour inoculum (*i.e.* whether freshly excised tumour of cultured tumour cells) and can be increased by administration of the adjuvant *C. parvum*. Furthermore, the tumour-associated Ig is heterogeneous in nature. Additional studies indicate that this binding cannot be attributed to a host response to endogenous C-type virus. Preliminary studies have also been performed to determine the cellular basis of this response and the specificity of the tumour-associated Ig.

**CELL-MEDIATED IMMUNE RESPONSE RELATED TO CARCINOMA OF THE CERVIX UTERI.** E. L. Murray, F. Sharp & C. T. C. Bowie, *Departments of Pathology and Gynaecology, University of Glasgow.*

We have used the leucocyte-migration-inhibition test as an index of cell-mediated immune response to material derived from carcinoma of the cervix and normal cervical epithelium, in groups of patients and control donors. These control donors comprised preoperative patients with benign gynaecological lesions and having a normal cervical smear. Higher reactivity to tumour-derived antigen was found in patients with invasive carcinoma of the cervix than in control donors. Reactivity with the same tumour extract in patients with preinvasive lesions of the cervix (carcinoma *in situ* and dysplasia) was less than that found in the benign control women. The diagnosis was established in each of the former by colposcopy and selective punch biopsy after collection of the blood for examination. In 4 cases with microinvasive lesions of the cervix, no reactivity was found.

**CIRCULATING IMMUNE COMPLEXES ASSOCIATED WITH GYNAECOLOGICAL CANCER.** P. J. McLaughlin,* M. R. Price*, R. W. Baldwin*, D. P. Vassey†, & E. M. Symonds†, **Cancer Research Campaign Laboratories, Nottingham, and †Department of Obstetrics and Gynaecology, City Hospital, Nottingham.*

We have tried to detect raised levels of circulating immune complexes in patients with ovarian cancer, using a variety of assays which are based upon interaction of complexes with components of the complement system. These assays include the direct Clq-immune complex precipitation test as described by Höffken *et al.*. 1977 (*Br. Med. J.*, ii, 218), the Clq-binding inhibition test (Fletcher & Lin (1977) *J. Immunol. Meth.*, **15**, 39) with which the capacity of native and decomplemented serum to inhibit the binding of $^{125}$I-Clq to IgG aggregates is measured, and the Raji cell-binding test (Theofilopoulos *et al.* (1976) *J. Clin. Invest.*, **57**, 169). With 8

ovarian cancer (Stage II–IV) patients, pre- and post-operative serum samples exhibited equivalent reactivity in these assays, and the immune-complex levels did not differ significantly from those of age-matched, non-malignant surgical control patients (8) or normal young female controls (25). In preliminary tests, elevated Clq-binding activity was however detected in patients (10/16) with other gynaecological tumours including carcinoma of the cervix and uterus as well as cervical dysplasia. These studies highlight the need for defining the malignant diseases in which measurement of immune complexes may prove of value in immunodiagnosis and prognosis.

**DETECTION OF FREE LIGHT-CHAIN IMMUNOGLOBULINS WITH CLINICAL POTENTIAL AS TUMOUR MARKERS.** D. F. Tucker, J. Keen & R. H. J. Begent, *ICRF, London and Department of Medical Oncology, Charing Cross Hospital, London.*

A method of detecting circulating immune complexes, which combines PEG precipitation, adsorption to *S. aureus* protein A and PAGE analysis of the fractionated material, was used to screen sera of patients with various types of malignant disease. To date, differences from the control pattern of protein bands produced by PAGE of fractionated normal sera, have been most readily obtainable with the cases of Hodgkin's and some other lymphomatous disorders. Judged by electrophoretic mobility and serological reactivity, an excess of apparently free immunoglobulin light-chain (LC) is present in a number of these patients' sera during active disease. Evidence will be presented showing the tendency for serum LC to return to normal levels during clinical improvement due to chemotherapy. This interesting relationship warrants further investigations of the potential value of serum LC monitoring in the clinical management of certain human lymphomas.

**THE Clq-BINDING TEST IN HUMAN BREAST CARCINOMA.** R. A. Robins, P. J. Doyle & R. W. Baldwin, *Cancer Research Campaign Laboratories, University of Nottingham.*

Previous studies in this laboratory have shown that the results of Clq-binding tests using plasma samples from primary breast-carcinoma patients correlate with other prognostic indicators, and outcome of disease at 2 years after mastectomy (Hoffken *et al.* (1978) *Lancet*, i, 672). Subsequent tests with serum samples have shown much lower levels of Clq-binding activity, and we have therefore investigated the influence of the method of sample collection on the results of Clq-binding tests. These studies have shown that the presence of heparin (used in our earlier plasma samples) results in a marked increase in Clq-binding activity of pathological serum samples, with little increase in control serum samples from healthy individuals. The presence of heparin also increases the Clq-binding activity of low levels of aggregated IgG. This modification of the Clq-binding test allows a clear difference between patients and controls to be demonstrated, and may be useful in determination of prognosis in primary breast carcinoma.

**LOCAL B-CELL IMMUNE RESPONSE IN BREAST CANCER; RELATIONSHIP WITH PRESENCE OF THOMSEN–FRIEDENREICH-ANTIGEN (TF).** G. G. Konorza, P. Müller, P. J. Klein & G. R. F. Krueger, *Pathology Institute, University of Cologne, West Germany.*

The role of B-cell-mediated immune response in tumour patients is unclear. This study attempts to correlate the amount and type (monoclonal/polyclonal) of Ig produced in the local inflammatory infiltrate around mammary ca. and in the regional lymph nodes with the presence of TF antigen on the tumour cells.

Routinely processed sections were examined in 19 cases, both tumour and lymph node. TF antigen was determined with marked lectins according to the method of Klein *et al.*, 1978 (*Klin. Wochenshr.*, **56**, 761). Local and lymphnode Ig production ($\delta$–, $\mu$–, $\lambda$–, $\kappa$–, chains in lymphoplasmocytoid cells) was assessed by immunofluoresence according to the method of Denk *et al.*, 1976 (*Bietr. Path.*, **159**, 219). It was found that Ig production is highest in differentiated carcinoma and here is mostly monoclonal. However, the amount of TF antigen correlates with the amount of Ig only in cases with a low or moderate expression of TF. Six cases with most Ig production showed little or no TF, the Ig synthesis being monoclonal in all 6. This suggests the existence of a strong antigen other than TF, the possibility of the "shedding" of TF-antibody complexes being an alternative, as shown by Nordquist *et al.*, 1977 (*Science*, **366,** 197) for other breast-cancer antigens. This possibility is supported by the findings of demonstrable amounts of Ig on tumour cells in one of these 6 cases.

**THE HISTOGENESIS OF NON-HODGKIN LYMPHOMAS ASSESSED BY SURFACE MARKING.** J. A. HABESHAW & A. G. STANSFELD, *ICRF Medical Oncology Unit, and Department of Pathology, St Bartholomew's Hospital, London.*

Surface marking of non-Hodgkin lymphomas shows that the phenotype of the neoplastic population corresponds to the phenotype of normal lymphocytes at different stages of differentiation. Both B and T lymphocytes are derived from a common stem cell which expresses ALL and Ia antigen. Tumours of this cell are rare (3%) and are always associated with ALL. Immature T-cell tumours have the phenotype HTLA$^+$ E rosette$^+$, and are TdT-enzyme positive. They form 4% of non-Hodgkin lymphomas. Subsets of mature T cells express receptors for IgG(Fc) and IgM (T$\gamma$ and T$\mu$ cells). In lymphomas, additional subsets of phenotype E$^+$C3$^+$ and E$^+$Fc$^+$IgM$^+$C3$^+$ have been identified and form 3% of NHL. The pre-B cell has cytoplasmic immunoglobulin (CyIg) but no surface immunoglobulin (SIg). It matures by an antigen-dependent pathway in marrow to the "virgin" immunocompetent B cell of SIg$^+$Fc$^+$IgM$^+$C3$^+$ phenotype acquiring Fc receptor (SIg$^+$Fc$^+$IgM$^+$) before C3 receptor (SIg$^+$Fc$^+$IgM$^+$C3$^+$). These phenotypes are only seen in CLL and in DWDL and form 11% of lymphomas. Subsequent differentiation is antigen dependent and is characterized by the formation of follicle-centre cells (SIg$^+$C3$^+$). Memory cells (SIg$^+$Fc$^+$C3$^+$), plasma-cell precursors (SIg$^+$CyIg$^+$) and immunoblasts (non-capping SIg$^+$) are derived from the follicular cell. The phenotype SIg$^+$C3$^+$ was found in 30% of cases. Non-capping SIg$^+$ cells formed 14% of lymphomas (mainly DHL) and pro-plasma cells 18% of lymphomas (DPDL). Memory B-cell tumours formed 7% of lymphomas (DPDL). 7% of lymphomas were formed of mixtures of B and T cells. The conclusion is that most non-Hodgkin lymphomas (79%) are derived from reactive lymphoid populations, and suggests that abnormal immune responses to undetected antigens are the major pathogenetic mechanism in lymphoma production.

**REED–STERNBERG CELL/LYMPHOCYTE INTERACTION. A NON-SPECIFIC ADHERENCE PHENOMENON.** S. V. PAYNE, D. G. NEWELL, D. B. JONES & D. H. WRIGHT, *University Department of Pathology, Southampton Medical School, Southampton General Hospital*

The dynamic interaction of T lymphocytes with the surface of isolated Reed–Sternberg (RS) cells may represent the immune T-cell attack on RS cells which has been postulated to be a central event in Hodgkin's disease (Order & Hellman (1972) *Lancet*, i, 571).

Ultrastructural studies have shown that this lymphocyte attachment ranges from point contacts by lymphocyte microvilli to close apposition of large areas of the two cell membranes, some stretches being regularly spaced at 10–15 nm apart. There were no membrane fusions, junctions, specializations of the underlying cytoplasm or lymphocyte invaginations, and no evidence of ultrastructural damage to the RS cells. RS cell/lymphocyte clusters may persist for 5 weeks in culture without loss of viability. Pharmacological studies have shown that this interaction is dependent on intact surface proteins (on both the lymphocytes and RS cells) and on divalent cations. It is independent of temperature, cell metabolism, intact microtubules and microfilaments and protein synthesis.

These features indicate that the attached T cells are not cytotoxic, and that this is a non-specific interaction unrelated to antigen-dependent immune adherence.

**CLASS-SPECIFIC ANTIBODY RESPONSES TO SURFACE ANTIGENS OF HERPES-INFECTED CELLS, IN PATIENTS WITH ABNORMAL CERVICAL CYTOLOGY AND PATIENTS WITH HERPETIC INFECTION.**

L. Mendis, J. M. Best & J. E. Banatvala, *Department of Virology, St Thomas' Hospital and Medical School, London.*

An indirect immunofluorescence test was used to detect IgG and IgA antibodies to surface antigens of cells infected with herpes simplex virus Type 2 (HSV-2). Sera were obtained from patients with invasive carcinoma from England, Malawi, Sri Lanka and Sudan, and from patients with dysplasia and carcinoma *in situ* from England. A significantly greater proportion of patients in all groups had IgA antibodies when compared with matched controls. There was also a marked geographical variation in both IgG and IgA antibody titres. IgA antibody titres in patients with abnormal cervical cytology from England were similar to those of patients with genital herpes (HSV-2) and were higher than those of patients with other malignancies. Other workers have reported no significant difference in the IgA titres to the intracellular virus-capsid antigen of HSV-2-infected cells between patients with invasive carcinoma and controls. Serial serum samples collected before and after radiotherapy showed a significant rise in geometric mean titre (GMT) of IgG antibodies and a fall in the GMT of IgA antibodies, although 4 patients from Malawi who were treated by hysterectomy showed no change in antibody titres. When sera were tested from patients with facial herpes (HSV-1) and genital herpes, the IgA antibody response appeared to be more type-specific than the IgG response.

### MONOCYTE COMPLEMENT RECEPTORS AND LUNG CANCER.

E. J. Glass & A. B. Kay, *Department of Pathology, University Medical School, Edinburgh.*

Chemotactic factors enhance the expression of complement receptors on human leucocytes, *i.e.* they produce an increase in the percentage of leucocytes which form rosettes with complement-coated red cells ("complement receptor enhancement"). The phenomenon was originally described with eosinophils and eosinophil chemoattractants (Anwar & Kay (1977) *Nature*, **269**, 522). Recently we have shown that complement receptors on neutrophils and monocytes were similarly enhanced. For instance, casein, a monocyte chemoattractant, enhanced monocyte C3 receptors in a dose- and time-dependent fashion. We have also studied the capacity of dialysates from human lung-cancer homogenates to inhibit enhancement of monocyte complement receptors ("enhancement-inhibition"). All the tumour-derived material so far examined produced "enhancement-inhibition" (4 undifferentiated, 4 squamous and 4 adenocarcinomas). The effects of the tumour-derived material were significantly greater than that of normal lung distant from the tumour. Preliminary studies suggest that the activity present in lung-cancer homogenates giving "enhancement-inhibition" is associated with a mol. wt of $\sim 8000$. These results support the view that tumours elaborate factors which inhibit their interactions with mononuclear phagocytes, an intimacy thought to be of importance in tumour surveillance (Supported by the Cancer Research Campaign)

### SURFACE AREA AND GROWTH CONTROL.

C. H. O'Neill, *Imperial Cancer Research Fund, London.*

A programmable calculator with digitiser input can be used to measure the exposed surface area of cultured cells, neglecting the small folds and projections which are not resolved by the light microscope. We have found that this area is related to serum concentration. Hamster fibroblasts show a 4-fold reduction in exposed area as the serum concentration is increased from 5% to 66%. At the higher concentration the mean exposed area of these cells is equal to the surface area of spherical cells, such as are seen in suspension culture. We have also found that the transition probability (a measure of growth rate) is more than 10 times greater in attached cells than in suspended cells, in 5% serum. At a serum concentration of 66%, the probabilities in attached and suspended cells become equal. It seems possible, therefore, that the only important difference between attached and suspended cells is the area of the surface exposed to the medium. Suspension inhibition, like density-dependent inhibition, may be a simple consequence of the rate of diffusion of serum growth factors.

**CYTOSKELETAL ELEMENTS AND GROWTH CONTROL: EFFECTS OF CYTOCHALASIN AND COLCHICINE ON 3T3 CELLS.** G. D. Clarke and P. J. Ryan, *Imperial Cancer Research Fund, London.*

BALB/c and Swiss 3T3 cells show an 8-fold difference in sensitivity to inhibition of DNA replication and thymidine-labelling index by cytochalasin B. They show a similar relative sensitivity to reduction in exposed surface area by the drug. These results, and observations of interacting effects of cell density, cytochalasin B and colchicine on BALB/c 3T3 cells, may be explained in terms of the exposed-surface-area model of growth control.

The involvement of microfilaments and microtubules in growth-control processes may be to alternatively restrict or optimize the exposed surface area of a cell and thus its interaction with macromolecular growth factors.

**INTERCELLULAR JUNCTIONS IN METHYLNITROSOUREA (MNU)-INDUCED CARCINOMA OF THE RAT URINARY BLADDER.** N. J. Severs & R. M. Hicks, *Cell Pathology Unit, School of Pathology, Middlesex Hospital Medical School, London.*

Alterations in intercellular junctions are associated with neoplastic transformation, and have been implicated in some of the biological properties of tumour cells (Weinstein *et al.*, 1976, *Adv. Cancer Res.*, **23,** 23). Using the freeze-fracture technique, we have investigated tight junction and nexus structure in normal Wistar rat urothelium, and in transitional-cell tumours Stage P1 (WHO classification) induced by intravesicular instillation of MNU. In normal urothelium, the zonula occludens consists of a network of 3–5 interconnecting fibrils encircling the apical lateral border of each superficial cell. Tumour-cell tight junctions have a markedly variable morphology; some are well developed and expanded to twice the normal width, whereas others are fragmented and may consist of only a few short isolated ridges. This change, also reported in FANFT-induced tumours (Merk *et al.*, 1977, *Cancer Res.*. **37,** 2843) may contribute to increased epithelial permeability. Maculae occludentes and composite occludens-nexus junctions are present deep within neoplastic but not normal urothelium. Two forms of nexus occur in normal tissue Type 1, composed of closely packed intramembrane particles (IMP), and Type 2, consisting of less compact larger IMP. In contrast to Pauli *et al.* (1977, *Lab. Invest.*, **37,** 609) we find both forms in tumours, although Type 1 does occur with greater frequency than in normal urothelium. These results suggest that in bladder cancer, abnormalities in growth control cannot be attributed to absence of nexuses. However, if differences in nexus morphology reflect functionally uncoupled and coupled states, the predominance of the Type 1 nexus in tumours might still indicate impairment of intercellular communication.

**MORPHOLOGY AND SURFACE PROPERTIES OF STEROID-RESPONSIVE AND UNRESPONSIVE MAMMARY-TUMOUR CELLS IN CULTURE.** J. Yates & R. J. B. King, *Imperial Cancer Research Fund, London.*

Cloned mouse mammary-tumour cells maintained in testosterone-containing medium ($^{+}$A cells) show a proliferative response to androgens which is lost after 3–5 weeks of culture in the absence of testosterone ($^{-}$A cells). Cytoplasmic and nuclear androgen receptors are present in the $^{-}$A cells. The $^{+}$A cells are fibroblastic in appearance, grow to high cell density forming multilayered foci and are able to grow in suspension. In contrast, $^{-}$A cells are epithelial and grow only as a monolayer. They show a higher rate of 2-deoxy-D-glucose uptake and a greater sensitivity to serum concentration than $^{+}$A cells. Plating efficiency of $^{+}$A but not $^{-}$A cells is diminished by concanavalin A. Scanning electron microscopy shows marked changes in the appearance and growth pattern of the cells after hormone deprivation. The morphological changes are being correlated with the loss of hormone responsiveness and changes in biochemical parameters.

**EFFECT OF GLUCOCORTICOIDS ON HUMAN ASTROCYTOMA: CELL ATTACHMENT PROLIFERATION AND TERMINAL CELL DENSITY *IN VITRO*.** R. I. Freshney, D. Morgan, M. Hassan-

ZADAH, M. BLACKIE & A. SHERRY, *Beatson Institute for Cancer Research, Bearsden, Glasgow G61.*

Stimulation of cloning efficiency and clonal growth was demonstrated when colonies were small, and was accompanied by a more compact colony morphology, implying greater cell–cell and cell–substrate adherence. When colonies reached 1000–2000 cells proliferation was reduced in treated colonies, producing greater uniformity in colony size than in controls, where a minority of colonies grow to a larger size than treated colonies.

Glucocorticoids reduced the lower terminal cell density of high-density multilayers and reduced incorporation of $^3$H-thymidine and lowered the labelling index. It appears that one effect of glucocorticoids on glioma is to promote cell adhesion and proliferation at initiation of clonal growth, but subsequently the effect on a high-density population is cytostatic.

Since glucocorticoids may stimulate cell adhesion by inducing synthesis of cell-surface glycoprotein, this may have led to better cell attachment and spreading, resulting in increased proliferation at low cell densities, while at high cell densities, density limitation of cell proliferation may be enforced by increased cell–cell adhesion.

**THICK HYALURONIDASE-SENSITIVE COATS ON TUMOUR CELLS.** W. H. McBRIDE & J. B. L. BARD, *Bacteriology Department, Edinburgh University Medical School & MRC Clinical & Population Cytogenics Unit, Western General Hospital, Edinburgh.*

A variety of adherent sarcoma, carcinoma and normal cells are surrounded by thick coats *in vitro* (around 9 $\mu$m thick) that can prevent spleen cells, as well as a variety of other cells and particles, from coming near to the cell membranes. This was obvious from the presence of large transluscent halos around the cells, which the indicator particles could not enter. Seven lymphoblastoid cell lines failed to show halos.

The presence of the thick coats of fibrosarcoma cells appeared to protect these cells from lymphocyte-mediated cytolysis. Hyaluronidase treatment, which destroyed the halo and allowed lymphocytes to approach the tumour-cell membrane, enhanced the cytotoxic action of immune but not of normal spleen cells. These findings may be relevant to the *in vitro* and *in vivo* killing of tumour cells by immune effector cells.

**SURFACE PROPERTIES OF LYMPHOID CELLS IN SPONTANEOUS RETICULUM-CELL SARCOMA (RCS) FROM SJL/J MICE.** F. ROBERT & F. DUMONT, *INSERM, Unit of Experimental Cancerology and Radiobiology, Vandoeuvre-lès-Nancy, France.*

SJL/J mice spontaneously develop RCS in abdominal lymph nodes. Studies on *transplanted* RCS have shown that the tumour cells carry $Ia^3$ antigen and exert stimulatory activity upon syngeneic T cells *in vitro* (Ponzio *et al.*, 1977, *J. Exp. Med.*, **146,** 132). However, little is known of the cellular events which occur in *primary* lesions. As an attempt to monitor the histogenesis of such lesions, we have investigated the electrokinetic (surface charge) and antigenic properties of individual cells from mesenteric lymph nodes (MLN) during the development of spontaneous RCS in 10–14-month-old SJL/J mice. In the early stages of MLN enlargement a bimodal electrophoretic distribution was regularly observed, with a low mobility (LM) peak corresponding to $SIg^+$, $Ia^+$, Thy $1\text{–}2^-$ B cells (35%) and a high mobility (HM) peak corresponding to Thy $1\text{–}2^+$, $SIg^-$, $Ia^-$ T cells (65%). As the cellularity of MLN increased, there was first an increase, then a decrease in the frequency of LM cells. In the ultimate stages of MLN hypertrophy, the bimodal pattern was replaced by a single electrophoretic peak in the HM region. Size analysis revealed the existence of at least 2 physical types of cells with lymphoid morphology. Population 1 which was predominant (80%) and had a modal volume of 165 $\mu m^3$, was characterized as Thy $1\text{–}2^+$, Lyt $1\text{–}2^+$, $Ia^{s-}$ and $sIg^-$. Population 2, of lowest surface charge (intermediate between typical B- and T-cells) had a modal volume of 300–350 $\mu m^3$. This latter population, which was $Ia^{s+}$ and $sIg^-$, was found markedly enriched in a transplantable RCS line grown *in vitro* and thus probably represents malignant elements. These data suggest that MLN hypertrophy in primary SJL/J lymphoma mainly results from a massive *in situ* proliferation of Lyt $1\text{–}2^+$ T cells possibly reflecting an auto-immune reaction against $Ia^{s+}$ malignant cells.

(Supported by Ligue Nationale Française Contre le Cancer.)